# Consensus-based technical recommendations for clinical translation of renal ASL MRI

**DOI:** 10.1007/s10334-019-00800-z

**Published:** 2019-12-12

**Authors:** Fabio Nery, Charlotte E. Buchanan, Anita A. Harteveld, Aghogho Odudu, Octavia Bane, Eleanor F. Cox, Katja Derlin, H. Michael Gach, Xavier Golay, Marcel Gutberlet, Christoffer Laustsen, Alexandra Ljimani, Ananth J. Madhuranthakam, Ivan Pedrosa, Pottumarthi V. Prasad, Philip M. Robson, Kanishka Sharma, Steven Sourbron, Manuel Taso, David L. Thomas, Danny J. J. Wang, Jeff L. Zhang, David C. Alsop, Sean B. Fain, Susan T. Francis, María A. Fernández-Seara

**Affiliations:** 1 Developmental Imaging and Biophysics Section, UCL Great Ormond Street Institute of Child Health, London, UK; 2 Sir Peter Mansfield Imaging Centre, School of Physics and Astronomy, University of Nottingham, Nottingham, UK; 3 Department of Radiology, University Medical Center Utrecht, Utrecht University, Utrecht, The Netherlands; 4 Division of Cardiovascular Sciences, School of Medical Sciences, Faculty of Biology, Medicine and Health, University of Manchester, Manchester, UK; 5 Translational and Molecular Imaging Institute and Department of Radiology, Icahn School of Medicine at Mount Sinai, New York, NY USA; 6 Department of Radiology, Hannover Medical School, Hannover, Germany; 7 Departments of Radiation Oncology, Radiology, and Biomedical Engineering, Washington University in St. Louis, St. Louis, MO USA; 8 Department of Brain Repair and Rehabilitation, UCL Queen Square Institute of Neurology, University College London, London, UK; 9 MR Research Centre, Department of Clinical Medicine, Aarhus University, Aarhus, Denmark; 10 Department of Diagnostic and Interventional Radiology, Medical Faculty, Heinrich Heine University Düsseldorf, Düsseldorf, Germany; 11 Department of Radiology and Advanced Imaging Research Center, UT Southwestern Medical Center, Dallas, TX USA; 12 Department of Radiology, Center for Advanced Imaging, NorthShore University Health System, Evanston, IL USA; 13 Imaging Biomarkers Group, Department of Biomedical Imaging Sciences, University of Leeds, Leeds, UK; 14 Division of MRI Research, Department of Radiology, Beth Israel Deaconess Medical Center and Harvard Medical School, Boston, MA USA; 15 Stevens Neuroimaging and Informatics Institute, University of Southern California, Los Angeles, CA USA; 16 A.A. Martinos Center for Biomedical Imaging, Massachusetts General Hospital, Harvard Medical School, Boston, USA; 17 Departments of Medical Physics, Radiology, and Biomedical Engineering, University of Wisconsin, Madison, Madison, USA; 18 Department of Radiology, Clínica Universidad de Navarra, Pamplona, Spain

**Keywords:** MRI, Arterial spin labelling, Kidney, Perfusion, Renal blood flow

## Abstract

**Objectives:**

This study aimed at developing technical recommendations for the acquisition, processing and analysis of renal ASL data in the human kidney at 1.5 T and 3 T field strengths that can promote standardization of renal perfusion measurements and facilitate the comparability of results across scanners and in multi-centre clinical studies.

**Methods:**

An international panel of 23 renal ASL experts followed a modified Delphi process, including on-line surveys and two in-person meetings, to formulate a series of consensus statements regarding patient preparation, hardware, acquisition protocol, analysis steps and data reporting.

**Results:**

Fifty-nine statements achieved consensus, while agreement could not be reached on two statements related to patient preparation. As a default protocol, the panel recommends pseudo-continuous (PCASL) or flow-sensitive alternating inversion recovery (FAIR) labelling with a single-slice spin-echo EPI readout with background suppression and a simple but robust quantification model.

**Discussion:**

This approach is considered robust and reproducible and can provide renal perfusion images of adequate quality and SNR for most applications. If extended kidney coverage is desirable, a 2D multislice readout is recommended. These recommendations are based on current available evidence and expert opinion. Nonetheless they are expected to be updated as more data become available, since the renal ASL literature is rapidly expanding.

**Electronic supplementary material:**

The online version of this article (10.1007/s10334-019-00800-z) contains supplementary material, which is available to authorized users.

## Introduction

Blood delivery to the tissue capillary bed (i.e. tissue perfusion) is critical to enable normal function, maintenance, and survival of physiological systems. In particular, the kidneys rely on a major, tightly regulated and continual supply of blood not only to remain viable but also to perform their key role in homeostasis, with filtration at the level of individual glomeruli at the forefront. Disruption of renal perfusion is linked to an array of pathophysiological mechanisms in acute kidney injury (AKI) as well as chronic kidney disease (CKD), with renal hypoperfusion (and subsequent hypoxia and fibrosis) involved in the former and potentially promoting progression of the latter [1–3]. As such, methods to accurately measure and monitor changes in renal perfusion would be of immense benefit to the clinic as well as allow novel investigations of renal function in vivo, particularly if able to do so at early stages of disease and in a safe and non-invasive way.

Arterial spin labelling (ASL), a magnetic resonance imaging (MRI) technique proposed over 25 years ago [4, 5], has arisen as one of the prime candidates for enabling an imaging-based quantification of tissue perfusion. To this end, arterial blood is used as an endogenous tracer, obviating the need for exogenous contrast agents. This particular feature of ASL, combined with the lack of need to employ ionizing radiation, renders it a fully non-invasive and universally applicable technique, regardless of age and degree of renal impairment, and highly suited for applications where serial monitoring of perfusion is required. Furthermore, ASL delivers the added benefit of being able to measure perfusion of each kidney separately, as well as assess intrarenal regional variations in perfusion. Its versatility makes it also perfectly feasible in kidney transplants, in spite of a very different vascular anatomy. Containing some of the most highly perfused tissue in the body, the kidneys were among the first organs where the feasibility of ASL to measure blood perfusion was reported [6]. However, in the first 20 years since those initial experiments, ASL was mostly confined to the brain, as susceptibility effects and motion limited its use in thoracic and abdominal organs. Nonetheless, work in renal ASL has substantially increased in the past few years, motivated by the interest in avoiding the use of gadolinium-based contrast agents, particularly in patients with significant renal impairment [7]. The last decade saw major technical developments which were accompanied by initial forays into several clinical applications. Many of these, as they pertain to kidney imaging in humans have been recently reviewed by the PARENCHIMA renal ASL expert panel [8] and by other authors [9, 10]. These reviews provide a compilation of published studies and a summary of data available on the validation and reproducibility of the technique for the assessment of renal perfusion in healthy and diseased kidneys. Concurrent with this growing interest, standardization of renal ASL methods is becoming crucial, particularly to enable large-scale multicenter studies and clinical trials. Nevertheless, the number of degrees of freedom in technical parameters of an ASL protocol is such that non-expert users are faced with a large array of choices which makes starting a study daunting. Furthermore, even though ASL acquisition methods are available in all major MRI scanner vendors, a lack of focus on renal imaging as a primary target often results in a limited capability of even state-of-the-art scanners to readily perform renal ASL using standard, commercially available pulse sequences.

We believe that providing clear technical recommendations for setting up data acquisition and analysis protocols will promote a widespread adoption of renal ASL and ultimately allow its potential for patient benefit to be realized sooner. This work aims to put forth a set of technical recommendations for renal ASL from an international group of experts working under the framework of the PARENCHIMA project, funded by a European Cooperation in Science and Technology (COST) Action (CA16103). One of our aims with these recommendations is to provide inexperienced sites with a robust starting point from which a robust renal ASL protocol can be developed with minimal replication of effort. Our hope is not for these recommendations to be seen as the definitive protocol for renal ASL but rather as a set of guidelines that capture the current consensus of a wide range of expertise in the renal ASL community. Rather than stifle innovation, we aim to stimulate clinical research using ASL by providing a straightforward set of parameters for image acquisition for non-specialist centers and facilitating the standardization of methods across different sites. Furthermore, we hope that by identifying what we hypothesize to be the current issues which require future investigation, we will inspire the scientific community to address these gaps in knowledge, focusing and accelerating worldwide efforts to progress renal ASL into a clinically useful technique, where it holds potential to improve the diagnosis and management of kidney disease.

### Previous literature review

One of the initial steps in the COST PARENCHIMA project included an extensive literature review of renal ASL [8]. This work summarized 53 studies on human subjects (excluding renal cancer) published until January 2018. Issues such as repeatability and validation of ASL with alternative methods to measure renal perfusion were extensively addressed therewith and, therefore, will not be covered in the present manuscript. The previous literature review [8] as well as 12 studies published in the intervening period [11–22], the results of a series of surveys and the discussions that took place at the in-person meetings form the basis for the final recommendations presented here.

### Variants of renal ASL and key parameters

**Fig. 1:**
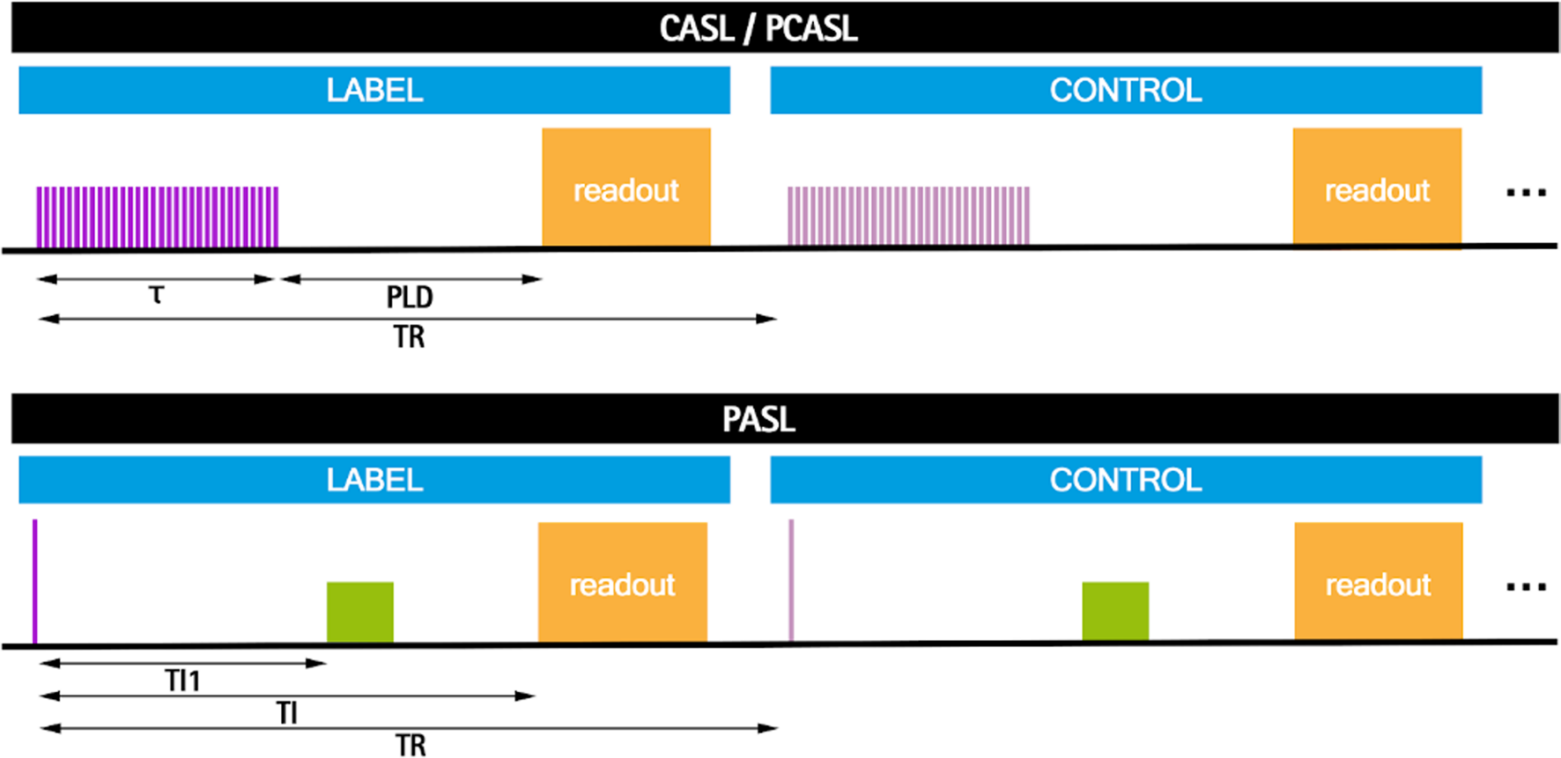
ASL timing diagram for CASL/PCASL and PASL, depicting both control and label conditions. Labeling pulses are shown in purple and the orange block represents the readout. TR is the repetition time. In CASL/PCASL: $\tau$ is the labeling time and PLD is the post-labeling delay. In the PASL scheme, the green block represents QUIPSSII type saturation pulses, TI1 is the bolus duration and TI is the inflow time

In ASL methods, radiofrequency (RF) pulses are employed to alter the longitudinal magnetization of blood water, rendering it a diffusible tracer. After allowing a time delay to ensure arrival of the “labelled” blood to the tissue microvasculature, a “label” (or “tag”) image is acquired. In the next repetition time (TR), the image acquisition is repeated without prior labelling of the inflowing blood, yielding a “control” image. In both measurements, the static tissue spins have experienced the same magnetization preparation. Therefore, its signal contribution can be removed by subtracting the two resulting images. However, the difference in signal due to inflowing blood (which has undergone different preparations) remains unaffected by the subtraction, and a perfusion-weighted image is obtained. In practice, the pair of label and control acquisitions is repeated to ensure sufficient signal-to-noise ratio (SNR). The ASL sequence can be thought of as the combination of two modules, a magnetization preparation module and a readout module, which are virtually independent. The magnetization preparation consists of inverting arterial blood magnetization before it reaches the tissue of interest. The effects of this inversion are detected by comparison with a control condition. This inversion can be realized using two main ASL variants: continuous and pulsed ASL (CASL and PASL, respectively) (see Fig. 1).

**Fig. 2:**
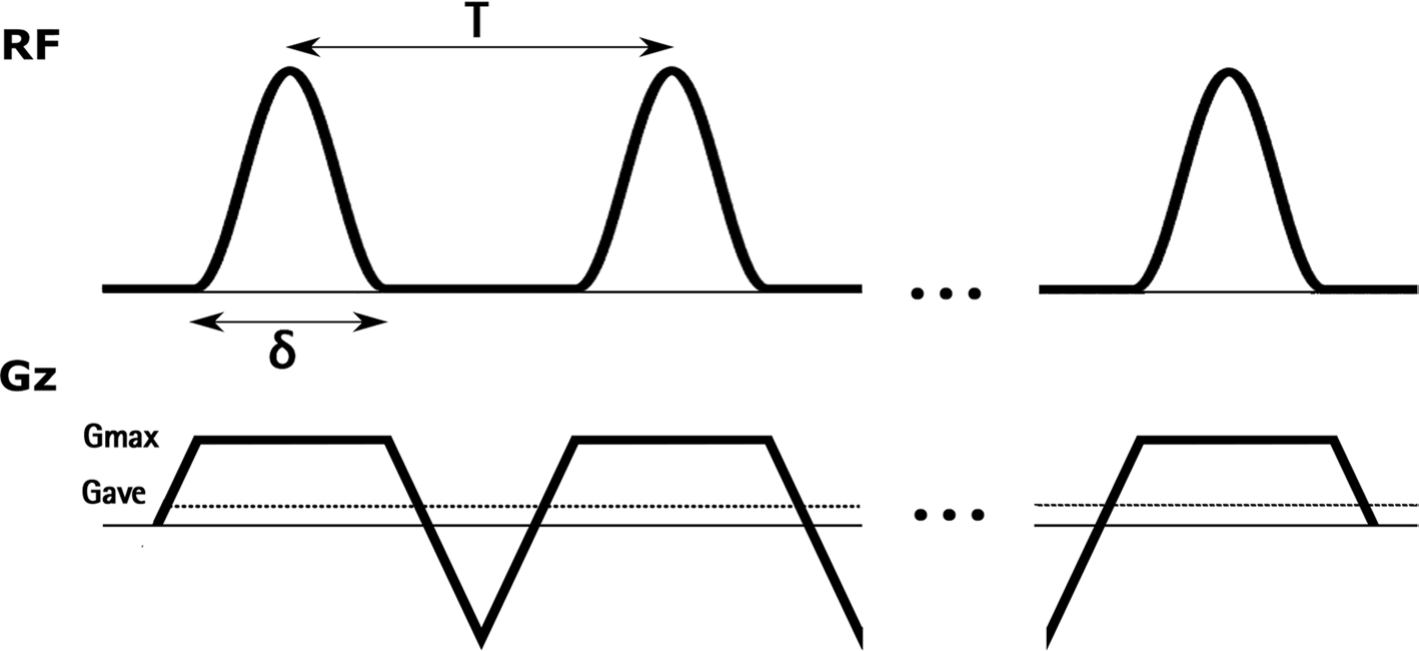
PCASL labeling scheme, showing successive RF pulses and gradients. $\delta$ is the RF pulse duration, $T$ is the RF pulse spacing, *G*_max_ is the amplitude of the slice selection gradient and *G*_ave_ represents the net average gradient

In CASL, blood magnetization is continuously inverted as it flows through a plane (the labelling plane). This continuous inversion is achieved by simultaneous application of a low-amplitude long-duration RF pulse and a gradient in the direction of flow, which causes the inversion of magnetization by adiabatic fast passage. Pseudo-continuous ASL (PCASL) [23] is a variant of continuous ASL in which the continuous RF pulse and gradient are replaced by a large number of selective RF pulses of short duration (see Fig. 2). In the gaps between successive RF pulses, the selective gradient (*G*_max_) is only partially rephased, resulting in a net average gradient (*G*_ave_) applied during the total duration of the labelling that mimics the continuous gradient applied in CASL. Due to the presence of this average gradient, the phase of successive RF pulses must be incremented by $\phi = \gamma G_{{{\text{ave}}}} {\text{TZ}}$, where $\gamma$ is the gyromagnetic ratio, $T$ is the RF pulse spacing and $Z$ is the position of the labelling plane with respect to the gradient isocenter so that they stay in phase with the flowing spins. During the control condition, the RF pulse train remains the same, thus cancelling magnetization transfer (MT) effects, except for the phase of successive RF pulses, which is incremented by $\pi$ radians. Two versions of PCASL have been proposed that differ in the control gradient waveform, named balanced and unbalanced PCASL. In balanced PCASL [24], the control gradient waveform is equal to that applied during the label, while in unbalanced PCASL [23], the selective gradient is totally rephased, resulting in a *G*_ave_ equal to zero. The duration of the RF pulse train is referred to as the labelling time ($\tau$), while the time interval from the end of labelling to the beginning of the readout is known as the post-labelling delay (PLD). PCASL has lower MT effects than CASL and can be used to efficiently image multiple slices simultaneously. In addition, it is compatible with the hardware available in most clinical MRI systems and has, therefore, replaced CASL, since its inception.

In PASL, the magnetization of a large volume of arterial blood is inverted almost instantaneously through the application of an inversion pulse. Multiple variants of PASL have been proposed; however, to date most renal ASL studies have used the flow-sensitive alternating inversion recovery (FAIR) approach [25, 26]. In FAIR, a non-selective adiabatic inversion centered at the imaging plane is applied for the label acquisition while for the control acquisition, a slab selective inversion inverts the magnetization in a narrow volume including the imaging slice. Since the only difference between the label and control conditions is the application of the selective gradient, FAIR is insensitive to MT effects. In FAIR, the time interval from the labelling pulse to the readout is termed the inflow time (TI). In order to obtain a quantitative value of perfusion using this approach with a single TI acquisition, the time duration of the labeled bolus must be known. This is achieved by applying saturation pulses to clearly define the bolus duration (using a QUIPSSII scheme, corresponding to the Quantitative imaging of perfusion using a single subtraction, version II method [27], or Q2TIPS scheme—QUIPSS II with Thin-slice TI_1_ Periodic Saturation [28]), in which saturation pulses are applied at time TI_1_ after the inversion pulse to destroy the tail end of the labeled bolus, thus effectively setting the bolus duration to be equal to TI_1_.

## Materials and methods

### Description of survey process

The Delphi method [29] constitutes a group effort to efficiently reach consensus on matters where quantitative evidence may be scarce, incomplete, or difficult to obtain. Details about the Delphi method can be found in numerous publications (e.g. [30, 31]) and further specific details about the consensus-generation approach used in the COST PARENCHIMA project can be found in Mendichovszky et. al. [32]. An in-person meeting in Prague preceded the formal start of the Delphi process allowing for attending panel members to start discussions and agree upon the method for generating recommendations [33]. The approach used for driving the consensus formation process included one initial preliminary survey and two “consensus” surveys [32, 34]. All surveys included questions focusing on obtaining technical recommendations to facilitate translation of renal ASL to the clinic and were divided into several categories: Patient preparation, Hardware, Labelling strategy, FAIR labelling parameters, PCASL parameters, Readout, Other sequence details, Data preprocessing and Quantification. The final survey (i.e. second consensus survey) additionally included a section on Data analysis/reporting. The preliminary survey [33] was set up with questions that allowed a combination of multiple-choice answers covering a broad range of settings for the main ASL parameters as well as open-ended questions. This allowed the panel co-chairs to draft an initial set of proposed statements already informed by the responses of the entire panel, which, to the extent possible, maximized the amount of initial consensus and minimized the number of further rounds necessary to refine statements for which consensus did not exist at the starting point.

**Fig. 3:**
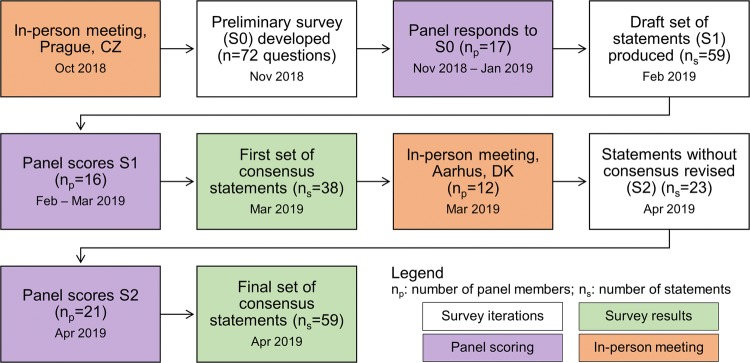
Overview of the consensus formation process

The first set of draft consensus statements was then shared with the entire panel for scoring, where the possible replies were: "1: Strongly Disagree", "2: Disagree", "3: Neutral", "4: Agree", "5: Strongly Agree" [33, 35]. Respondents were asked to provide further details when disagreeing with any particular statement [35]. Consensus was assumed to be found when 75% or more of respondents agreed with a particular statement. Neutral responses were considered abstentions and were excluded from the calculation. The second in-person meeting took place after the scoring of the first set of consensus statements to present results to the group and allow for discussion to modify/rephrase statements for which consensus had not been found [33–35]. Half of the respondents to the survey attended the second in-person meeting. The revised set of consensus statements was then rescored by the entire panel where the possible replies were: "I agree", "I disagree", "I have insufficient experience to make a recommendation" [34]. The latter was considered abstention and was removed from the consensus calculation. The threshold for consensus was also 75% (inclusive) of agreements. A summary of this process is shown in Fig. 3. The complete list of questions which composed each survey is provided as supplementary material.

### Panel characteristics

The panel comprised clinical and non-clinical researchers with expertise in renal ASL as evidenced by a previous track-record of publications and/or ongoing research in renal ASL, working in six countries and with backgrounds as follows: Engineer, 17%; Nephrologist, 4%; Physicist, 70%; Radiologist, 9% (background, percentage of total number of panel members; see Table 1). The degree to which panel members participated in the different stages of the Delphi consensus method is summarized in Table 1.

**Table 1:** Panel characteristics and participation in consensus process; 0: no; 1: yes; *S* <*n*> : survey number “*n*”; IPM2: second in-person meeting

Panel member (#)	Country	Background	S0	S1	IPM2	S2
1	US	Engineer	1	1	0	0
2	UK	Nephrologist	0	0	1	0
3	DE	Physicist	1	0	0	1
4	DK	Physicist	0	0	1	1
5	US	Physicist	1	1	0	1
6	UK	Physicist	1	1	1	1
7	US	Physicist	1	1	0	1
8	US	Physicist	0	0	0	1
9	UK	Physicist	0	0	0	1
10	US	Physicist	0	0	1	1
11	ES	Engineer	1	1	1	1
12	UK	Physicist	1	1	1	1
13	UK	Engineer	1	1	1	1
14	US	Physicist	1	1	0	1
15	NL	Engineer	1	1	1	1
16	US	Physicist	1	1	0	1
17	UK	Physicist	1	1	1	1
18	UK	Physicist	1	1	0	1
19	US	Physicist	1	1	0	1
20	DE	Radiologist	1	1	1	1
21	US	Physicist	0	0	1	1
22	US	Radiologist	1	1	0	1
23	US	Physicist	1	1	1	1

## Results

The response rate for the three surveys (preliminary + 2 consensus surveys) was 74%, 70%, and 91%. Some of the researchers joined the panel and took part in the process after the second in-person meeting, which explains the lower response rate of the first two surveys. For most of the consensus statements, the percentage of agreement was much higher than the pre-established threshold of 75%. Indeed, for 42% of statements the whole panel was in agreement. This shows that there is a clear convergence among experts regarding the basic technical aspects of renal ASL data acquisition and analysis. Overall, for the statements for which consensus was found the abstention and agreement level were 12 ± 10% and 94 ± 7%, respectively.

### Consensus statements

The 59 final consensus statements are listed in Table 2, as well as the abstention and agreement level for each statement.

**Table 2:** Final consensus statements

#	Statement	Abstentions (%)	Agreement (%)
*1. Patient preparation*			
1.1	Subject should be scanned in a normal hydration status when clinically appropriate	24	100
*2. Hardware*			
2.1	Both 1.5 T and 3 T are adequate field strengths	0	87
2.2	The body coil should be used as transmitter coil	6	93
2.3	Body phased-array coils should be used as receive coils	0	94
*3. Labeling strategy*			
3.1	Both PASL:FAIR and PCASL are adequate labeling strategies	6	93
3.2	Single time point acquisitions are recommended for simplicity of acquisition and data analysis	6	86
3.3	Multiple time point acquisitions require a longer acquisition time and more complicated processing. However, they can provide measurements of perfusion and ATT that can be useful if delayed arrival time is suspected in a clinical population	6	100
*4. FAIR labeling parameters*			
4.1	A FOCI pulse should be used for the selective inversion to optimize the inversion slice profile	19	92
4.2	The selective slab should be carefully positioned, excluding the aorta	6	100
4.3	The selective inversion slab thickness should equal the imaging slab thickness + [10–20] mm	13	86
4.4	In single-TI acquisitions, an inversion time of 1.8–2.0 s is recommended	10	89
4.5	In single-TI acquisitions, an approach for controlling the temporal width of the bolus (QUIPSS II or Q2TIPS) must be used to quantify perfusion	25	100
4.6	A bolus duration (TI_1_) of 1.0–1.2 s is recommended	25	92
4.7	In single-TI acquisitions, a minimum of 20 ASL pairs is recommended	10	89
*5. PCASL parameters*			
5.1	An unbalanced version of PCASL is preferred due to its lower sensitivity to off-resonance effects	38	80
5.2	A labeling time of 1.5–1.8 s is recommended	10	100
5.3	The labeling plane should be oriented approximately perpendicular to the aorta	13	100
5.4	The labeling plane should be positioned at approximately 8–10 cm from the centre of the kidney, in the superior direction	14	94
5.5	Hanning RF pulses are recommended	31	100
5.6	An RF pulse duration of 500 μs is recommended	25	100
5.7	Pulse spacing (from the centre of one pulse to the centre of the next) of 1000 μs or shorter is recommended	31	100
5.8	Average B_1_ of 1.6 μT is recommended	27	100
5.9	Average gradient (*G*_ave_) of 0.4–0.6 mT/m is recommended	25	92
5.10	*G*_max_ to* G*_ave_ Ratio of 6–7 is recommended	40	100
5.11	In single PLD acquisitions, a PLD of 1.2–1.5 s is recommended	19	100
5.12	In single-PLD acquisitions, a minimum of 20 ASL pairs is recommended	14	83
*6. Readout*			
6.1	A 2D single-slice acquisition scheme is recommended as the default renal ASL method	10	95
6.2	Multislice 2D acquisition schemes are recommended for applications that require extended kidney coverage	5	100
6.3	3D acquisition schemes represent a promising alternative to 2D multislice schemes but are not recommended as the default method for extended kidney coverage due to limited clinical experience with 3D schemes	10	95
6.4	Spin-echo EPI is the preferred readout for 2D single-slice acquisitions	5	75
6.5	bSSFP and single-shot RARE are adequate alternatives to EPI for 2D single-slice acquisitions	14	94
6.6	Spin-echo EPI is the preferred readout for 2D multislice acquisitions	14	83
6.7	Coronal oblique slices (along the major axis of the kidneys) are preferable for renal ASL	6	93
6.8	The recommended slice thickness in 2D acquisitions is 4-8 mm	19	100
6.9	The recommended slice thickness in 3D acquisitions isss 3-6 mm	13	100
6.10	The recommended in-plane resolution is 2-4 mm	0	93
6.11	Undersampling methods, such as partial Fourier and parallel imaging at moderate acceleration factors (up to R = 2) may be used	19	100
6.12	The recommended TR (including labeling + readout) is 4–6 s	0	94
*7. Other sequence details*			
7.1	Pre and post-inversion saturations are recommended for FAIR labeling schemes	14	100
7.2	Background-suppression is recommended for renal ASL	5	80
7.3	Breath-hold scans are not recommended for clinical renal ASL	0	94
7.4	Renal ASL scans under free breathing are preferred	0	76
7.5	Respiratory triggering can be advantageous to minimize the effects of kidney motion at the expense of scan time	5	95
7.6	Fat suppression is recommended for renal ASL	5	90
*8. Data preprocessing*			
8.1	Retrospective image registration is highly recommended for renal ASL	13	100
8.2	Outlier rejection is recommended for renal ASL	0	100
*9. Quantification*			
9.1	M_0_ acquisition is mandatory	0	94
9.2	Using a single-compartment model with assumed blood T_1_ for quantification is recommended for robustness and simplicity	7	100
9.3	A two-compartment model with separate transit time and tissue T_1_ measurements is a viable alternative to the single-compartment approach but requires more complex acquisition/analysis methods and therefore is currently not recommended as the default renal ASL approach	10	95
9.4	Tissue-blood partition coefficient = 0.9 mL/g [82–84, 97]	5	90
9.5	Assumed blood T_1_ at 3 T = 1.65 s [98]	0	100
9.6	Assumed blood T_1_ at 1.5 T = 1.48 s [98]	13	93
9.7	Labeling efficiency PASL = 95% (neglecting background suppression loss)	6	100
9.8	Labeling efficiency PCASL = 85% (neglecting background suppression loss)	13	86
9.9	When background suppression is used, the labeling efficiency needs to be adjusted based on the number of background suppression pulses	19	100
9.10	Regions of interest selection should be performed manually as the default approach. Semi-automatic methods may be used if local expertise is available (e.g. using T_1_ maps) but require extensive validation	0	100
9.11	Region of interest selection should be performed based on the ASL M_0_ image or a separately acquired structural dataset	6	93
*10. Data analysis/reporting*			
10.1	Cortical renal blood flow values (not whole-kidney) should be reported, separately for left and right kidney	0	100
10.2	Medullary renal blood flow values are not considered reliable with current measurement approaches	14	89

**Fig. 4:**
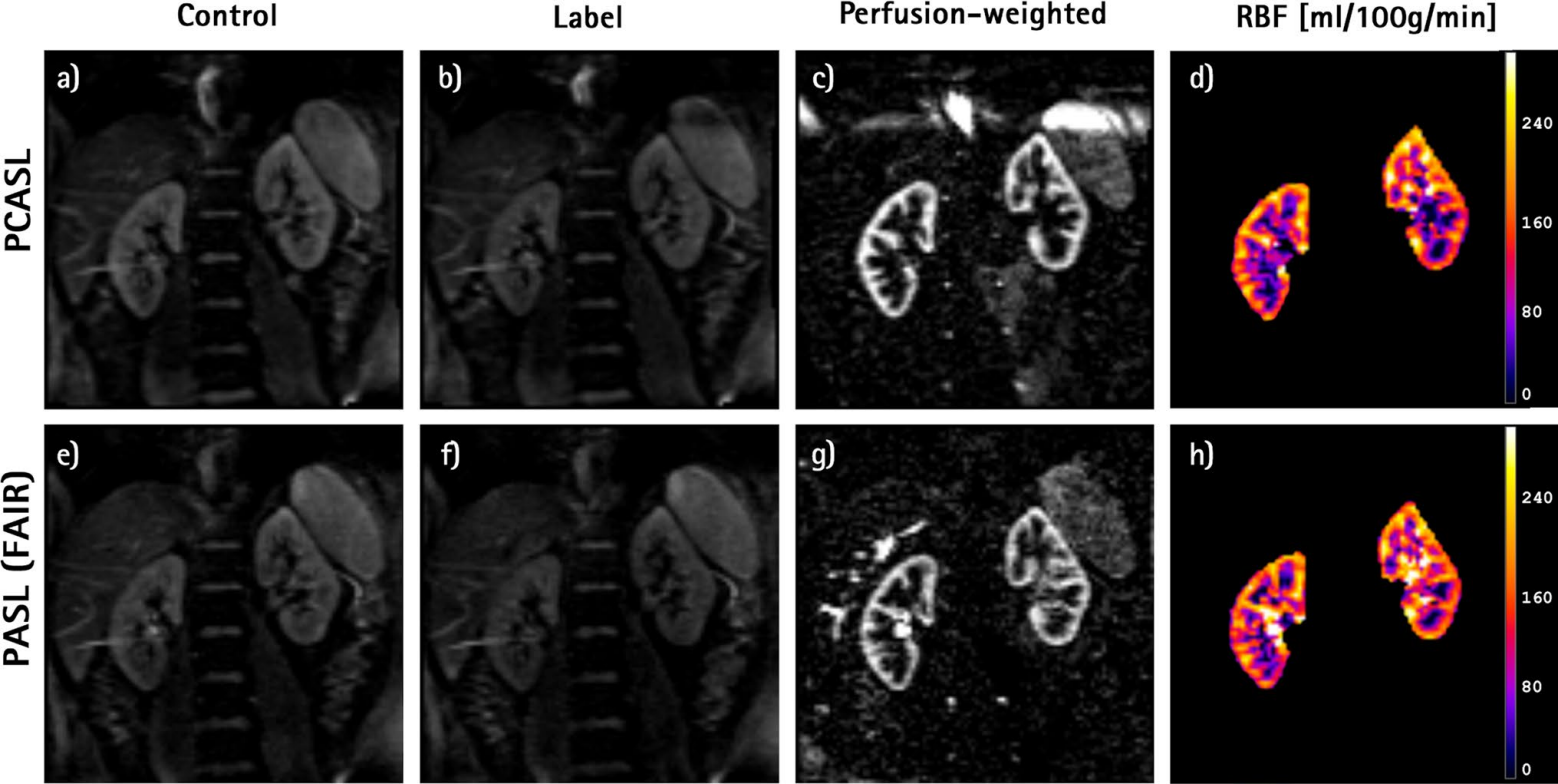
Example of renal ASL data from a healthy volunteer acquired at 3 T using the recommended protocol. Top row images were obtained with PCASL and bottom row with PASL (FAIR) labeling: **a**, **e** control; **b**, **f** label; **c**, **g** mean perfusion-weighted image; **d**, **h** RBF map. Data were acquired using background suppression (3 pulses), followed by a SE-EPI readout

In summary, as a default implementation, we recommend acquisition of coronal oblique ASL data at 1.5 T or 3 T using PASL (FAIR) or PCASL labelling at a single time point and using a 2D readout with background suppression. Examples of renal ASL images acquired from a healthy volunteer using such a protocol are shown in Fig. 4, together with the corresponding RBF map. Furthermore, examples of renal ASL data obtained in three patients with CKD can be seen in the supplementary material (Figure S1).

### Lack of consensus

The statements for which consensus could not be reached are listed in Table 3. Both statements concerned patient preparation by dietary restrictions and are indirectly related to the technical setup of renal ASL protocols, the main area of focus of these recommendations.

**Table 3:** Statements for which consensus was not found

#	Statement	Abstentions (%)	Agreement (%)
*1. Patient preparation*			
A	Diet needs to be controlled before the scan	43	50
B	Subjects are required to follow a controlled and standardized salt intake before the scan	48	27

## Discussion

In this section, recommendations are numbered and can be identified by the prefix "*R*".

### Patient preparation

The panel agreed that the subject should be scanned in a normal hydration state where appropriate **[R1.1]**. Measuring hydration status is challenging and there is no universally accepted reference standard measure [36, 37]. Standardizing patient preparation with the aim of achieving more reproducible renal perfusion measurements may be desirable. Renal ASL perfusion estimates have shown significant variation when subjects were challenged with water loading, protein load, adenosine, and furosemide [20, 38–40].

The large abstention level on some aspects related to patient preparation (see Table 3) may reflect uncertainty and a lack of definitive evidence in the literature with regard to these issues. It might also reflect the large variety of experts, not all nephrologists, and, therefore, not all capable of recommending on such aspects of patient preparation.

### Hardware

The panel agreed that both 1.5 T and 3 T are adequate field strengths for renal ASL measurements **[R2.1]**. Imaging at 3 T provides inherently higher SNR, which in ASL is further boosted by longer longitudinal relaxation times of the ASL label and renal tissue. However, susceptibility-induced B_0_ inhomogeneity is more pronounced increasing the propensity to artefacts such as distortion and signal loss. Shorter *T*_2_* at 3 T compared to 1.5 T further contributes to signal loss. In addition, greater power deposition (which increases with the square of the B_0_ field strength) may pose slice coverage limits particularly in sequences with more intensive use of RF energy. Results of published studies in which renal ASL measures have been collected at field strengths of 1.5 or 3 T show a similar range of renal cortex perfusion values in healthy subjects and in renal disease and similar reproducibility [41, 42]. The panel agreed that a system-integrated body coil should be used for RF transmission **[R2.2]** together with body phased-array receive coils **[R2.3]**.

### Labelling strategy

A consensus was reached that both pulsed ASL FAIR and PCASL schemes are adequate for renal ASL **[R3.1]**. For each, single time point acquisitions, defined to be at a given TI for FAIR and PLD for PCASL, are recommended for simplicity of acquisition and data analysis **[R3.2]**. Multiple time point acquisitions, in which data are collected at a number of TIs [20, 43, 44] or PLDs [45], require a longer acquisition time and more complicated processing. However, it was agreed that a multiple time point method can be useful if delayed arrival time is suspected in a clinical population, as this provides a measurement of arterial transit time (ATT) which can facilitate more accurate quantification of renal perfusion **[R3.3]**.

### FAIR labelling parameters

**Fig. 5:**
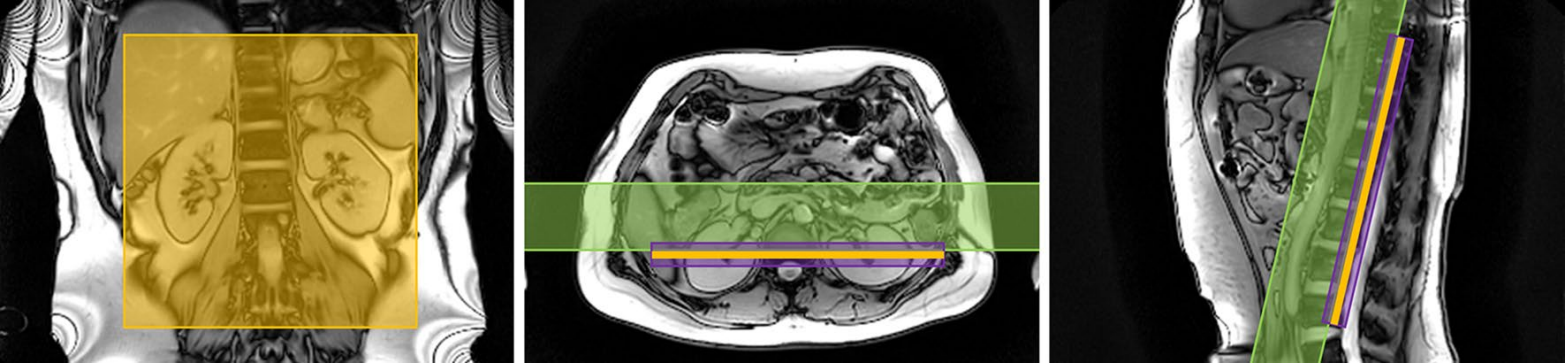
Positioning of the FAIR selective inversion slab (in purple), imaging slice (in orange) and QUIPSSII/Q2TIPS type saturation slab (in green) is shown on anatomical localizers in the coronal, axial and sagittal orientations

In FAIR, control and label conditions are achieved by alternating between a non-selective and selective inversion slab. The RF pulse used for the selective inversion should have sharp inversion profile edges, and an adiabatic frequency offset corrected inversion (FOCI) pulse [46, 47] is recommended for this purpose **[R4.1]**. The selective inversion slab is placed in-line with the imaging slab and should be carefully positioned to exclude the feeding arteries (descending aorta for native kidneys) to avoid labelling of blood in the control condition resulting in the maximum signal difference between label and control conditions due to inflowing blood (i.e. to maximize perfusion signal) **[R4.2]** (see Fig. 5). The thickness of the selective inversion slab should equal the imaging slab with an addition of 10–20 mm to avoid a mismatch between the location of the selective inversion slab and imaging slab (e.g. due to motion in the time (TI) between labelling and readout) **[R4.3]**. The choice of added thickness is determined particularly by the available space between the selective inversion slab and the aorta which is also dependent on the number of imaging slices being acquired. A TI of 1.8–2.0 s is recommended in single-TI acquisitions **[R4.4]** to allow sufficient time for the labeled blood to arrive at and exchange with the renal parenchyma (see Figure S2 in the supplementary material). Previous studies in healthy volunteers have mostly used somewhat shorter TIs, but studies in patients often have used a longer TI of ~ 2 s [19, 48, 49]. At 1.5 T the more rapid T_1_ decay of the label should be taken into account in the choice of TI. To quantify renal blood flow, the temporal width of the bolus should be defined. This can be achieved by the application of saturation pulses (QUIPSSII [50] or Q2TIPS [28]) at the location of the feeding arteries with a certain delay time after the FAIR labelling pulse (referred to as bolus duration or TI_1_) **[R4.5]**. A bolus duration (TI_1_) of 1.0–1.2 s is recommended **[R4.6]** to allow sufficient labelled blood to enter the tissue before the tail of the bolus is saturated such that it no longer contributes to the perfusion signal. To improve SNR, multiple ASL pairs (control and label images) are acquired during a scan. The acquisition of a minimum of 20 ASL pairs (control and label images) when using the recommended 2D readout is advised to obtain an averaged perfusion weighted image with sufficient SNR **[R4.7]**.

### PCASL parameters

The PCASL parameters recommended here have been chosen with the objective of maximizing labelling efficiency while increasing the robustness of the labelling in the presence of magnetic field B_0_ variations over the region of the labelling plane. Both versions of PCASL (balanced and unbalanced) have been successfully employed in renal ASL; however, recent work has demonstrated greater robustness of unbalanced PCASL to off-resonance effects [51]. Although this work was done in the context of brain ASL, the same results would be expected when labelling the aorta, where magnetic field variations are likely to be more pronounced than in the carotid arteries, due to the proximity of the lungs and vertebral column. Although both approaches have not yet been directly compared in the kidney extensively, the unbalanced implementation of PCASL is considered preferable to the balanced implementation [52], if available **[R5.1]**. The efficiency of PCASL increases with the average B_1_ amplitude of the labelling RF pulses; however, in practice the B_1_ amplitude is restricted by specific absorption rate (SAR) limitations. The recommended value of 1.6 µT should provide high efficiency while keeping SAR within acceptable safety limits **[R5.8]**. It is worth emphasizing that this value represents the average value over the time (see Fig. 2), not the RF pulse peak amplitude. Hanning-shaped RF pulses of 500 µs duration are recommended to improve the spatial selectivity of the RF pulse train **[R5.5, R5.6]**. The pulse spacing ($\it{T}$) (time from the center of one pulse to the center of the next) should be kept as short as possible to decrease the sensitivity to resonance frequency offsets. Due to duty cycle constraints, in most scanners the pulse spacing cannot be shorter than 1 ms **[R5.7]**. *G*_ave_ values of 0.4 to 0.6 mT/m are recommended **[R5.9]**. These values maximize labelling efficiency for the pulsatile velocity waveform characteristic of blood flow at the abdominal aorta [53]. A ratio of selective to average gradients, *G*_max_/*G*_ave_, of 7 is recommended **[R5.10]**. This ratio, lower than previously proposed for brain ASL [54], has been shown to improve off-resonance labelling [51]. However, a lower ratio results in a wider labelling region and, therefore, care must be taken when positioning the labelling plane (as discussed next).

**Fig. 6:**
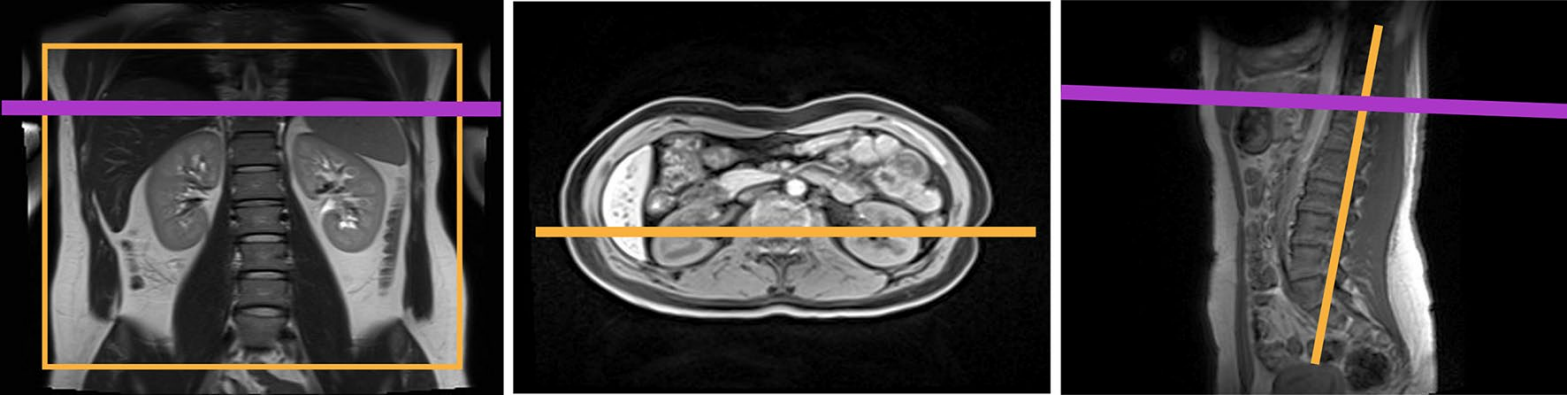
Positioning of the PCASL labeling plane (in purple) and imaging slice (in orange) is shown on anatomical localizers in the coronal, axial and sagittal orientations

The labelling plane should be positioned carefully, oriented approximately perpendicular to the aorta **[R5.3]** (see Fig. 6), and positioned above the highest kidney (to prevent direct saturation), while avoiding intersecting the heart. If allowed by anatomical constraints, it should be placed below the lungs to minimize off-resonance effects. A distance of 8–10 cm from the center of the highest kidney is recommended to fulfil these conditions in most cases **[R5.4]**, although the optimal position will be determined by the specific patient size and anatomy.

A labelling duration of 1.5–1.8 s is recommended **[R5.2]** [11, 21, 55–59]. Longer labelling durations would provide higher SNR for a given image, but would also lengthen the TR, reducing the number of averages acquired per unit time and increasing the power deposition. A PLD of 1.2–1.5 s is recommended in single PLD acquisitions **[R5.11]** [11, 21, 55, 57–60]. This PLD should allow for all the labeled blood to arrive at the renal parenchyma before image acquisition in most subjects, thus providing perfusion weighted data which can be quantified to yield quantitative renal blood flow values. This PLD is longer than the analogous quantity TI–TI_1_ in PASL to compensate for the longer transit delay in PCASL compared to PASL [50]. For some patient populations, where ATT could be abnormally long (such in the case of renal artery stenosis) a longer PLD could be desirable; however, lengthening the PLD would also lead to decreased SNR due to T_1_ decay. Finally, the acquisition of a minimum of 20 ASL pairs (control and label images) when using the recommended 2D readout is advised to obtain an average perfusion weighted image of sufficient SNR **[R5.12]**.

### Readout

An important consideration for renal ASL is the choice of readout scheme. The intrinsically low SNR of ASL necessitates high SNR readout schemes. However, due to the rapid decay of the ASL signal, the readout time must be short. The optimal ASL readout scheme should have a short echo time (TE) in order to provide the highest image SNR and reduce the amount of signal loss and distortions. Short image acquisition times are particularly important in multislice acquisitions, so that a short temporal spacing can be achieved between multiple slices allowing the volume to be acquired prior to the decay of the ASL signal.

The first consideration is whether a single or multislice readout scheme should be used. A consensus was reached that a single-slice acquisition scheme is recommended as the default renal ASL method **[R6.1]**. This recommendation was motivated by the robustness and reproducibility of single-slice acquisitions, which are easier to plan, especially when combined with FAIR labelling, and the fact that background suppression of the static tissue can be optimized for a single time point only. Moreover, acquisition of single-slice perfusion data was considered sufficient in most pathologies where kidney dysfunction is widespread and affects the whole kidney in a uniform manner. Exceptions to this would be cases in which renal dysfunction is due to unilateral and/or focal lesions due to vascular, tumoral, obstructive or infectious causes. Examples include certain causes of acute kidney injury such as sepsis and renal papillary necrosis caused by nonsteroidal inflammatory drugs [61]. In cases such as these where extended kidney coverage is required, it is recommended to use a multislice 2D acquisition scheme [12] **[R6.2]**, although in this case, the degree of background suppression will vary across slices. It was recognized that 3D acquisitions represent a promising alternative to 2D multislice schemes that can be optimally combined with BGS [14, 21], but are at this time not recommended as a default method due to limited clinical experience with these techniques **[R6.3]**.

For a 2D readout scheme, this survey asked participants to choose between balanced steady-state free precession (bSSFP) [62], spin-echo echo-planar imaging (SE-EPI) [63], and single-shot rapid acquisition with relaxation enhancement (RARE) [55, 64]. Gradient-echo echo planar imaging (GE-EPI) was not considered because of its susceptibility to the magnetic field inhomogeneities that are present in abdominal regions. A bSSFP readout has the advantage of high image SNR and a very short echo time (TE) but has a long image acquisition time and temporal image spacing due to SAR constraints. This limits the number of slices that can be acquired before signal decay of the label, rendering bSSFP suboptimal for applications where multiple slices are required. A bSSFP scheme is also sensitive to field inhomogeneity, which results in banding artefacts in areas of off-resonance within the image. A SE-EPI sequence has the advantage of rapid acquisition allowing multiple slices to be acquired at the peak of the ASL signal curve leading to low variance in the perfusion-weighted signal across slices. However, due to the large field of view (FOV) generally used in body imaging, EPI readouts can have a long echo time (TE) which limits their use for high-resolution imaging. SE-EPI readouts are also susceptible to chemical shift artefacts (e.g. from fatty tissue) (see Figure S2 in the supplementary material). A RARE readout scheme provides high image SNR and has low susceptibility-induced signal loss. However a 2D RARE readout has a long TE, echo train duration and high SAR due to the multiple refocusing pulses. This results in a long temporal slice spacing for multislice imaging, limiting the number of slices achievable at the peak of the ASL signal. Taking into consideration the aforementioned reasons, and the widespread availability across scanner vendors, a consensus was reached that SE-EPI is the preferred readout scheme for 2D single-slice imaging **[R6.4]** and multislice **[R6.6]** acquisitions, with bSSFP and single-shot RARE considered adequate 2D single-slice alternatives **[R6.5]**. A consensus was reached that coronal oblique slices (along the major axis of the kidneys) are preferable for renal ASL **[R6.7]**. For FAIR, this is necessary to avoid labelling of the inflowing vessels for the selective label. A coronal oblique orientation also has the advantage that most kidney movement due to the respiratory cycle is within the image plane and so data can be corrected using image registration methods. In addition, if full coverage is required, fewer slices are needed in coronal oblique scans.

For a 2D acquisition, the recommended slice thickness is 4–8 mm **[R6.8],** whereas for a 3D readout the recommended slice thickness is 3–6 mm **[R6.9]**. Consensus was reached for an in-plane resolution of 2–4 mm **[R6.10]**. The use of undersampling methods such as partial Fourier and parallel imaging is recommended to reduce TE and readout time and thus minimize susceptibility-induced geometric distortions, signal loss and blurring. Low acceleration factors up to 2 are preferred because higher acceleration leads to SNR loss which is not desirable since ASL is SNR-limited **[R6.11].** The recommended TR (including labelling and readout) is 4–6 s **[R6.12]**.

### Other sequence details

Throughout the development of ASL, a number of pulse sequence modifications have been introduced that aim to improve perfusion measurements by minimizing systematic errors that introduce variance in the ASL difference signal unrelated to tissue perfusion. Such errors can be caused by imperfect inversion profiles between the label and control in the FAIR scheme and/or MT effects of the labelling on the tissue in the imaging slice. These errors can be addressed by applying pre- and post-saturation pulses to the imaging volume [65, 66], and their use is recommended for FAIR **[R7.1]** and can also be applied to reduce the background signal variance in PCASL. ASL is challenged by the fact that the proportion of perfusion-weighted signal from inflowing labelled blood is low (on the order of 5% in healthy kidneys) compared to the static tissue signal. As such, fluctuations in the static tissue signal can overwhelm the perfusion-weighted signal and thus bias perfusion estimates. Background suppression (BGS) was proposed to reduce signal from static tissue and thereby the deleterious effects of these fluctuations [67]. This is achieved with the use of multiple carefully timed inversion pulses to significantly reduce the longitudinal magnetization of static tissue at the instant of data acquisition [68]. BGS is used widely and is currently recommended for ASL in neuroimaging [54]. In the kidneys, initial studies by Robson et al. [55]. and Gardener et al. [63] demonstrated an improvement of ASL image quality when using BGS. However, the latter reported that in the body, in the presence of respiratory motion, this improvement was at the expense of SNR when compared to using image registration. Nevertheless, recent studies have demonstrated the capability of BGS to improve the temporal signal-to-noise ratio (tSNR) and image quality of ASL data and shown image registration to be effective even in data acquired with heavy BGS [11, 21]. For these reasons, we recommend the use of BGS for renal ASL **[R7.2]**, and generally 2 to 4 BGS adiabatic inversion pulses have been employed in previous studies. It is worth noting that any inefficiency in the inversion pulses used in the BGS scheme reduces the ASL difference signal [69] which should be accounted for in the quantification stage (see below) and suggests a balance has to be struck between the number of BGS pulses used for effective reduction of static tissue signal fluctuations and labelling efficiency.

Subject breathing induces kidney displacements up to an order of magnitude larger than the typical ASL voxel size, which if unaccounted for cause a significant loss of image quality in ASL. Breath-holding effectively reduces kidney motion due to respiration but to compensate for the intrinsically low SNR of ASL measurements, acquisitions require either numerous or lengthy breath-holding periods, which can be challenging for patient populations [70–72] and even healthy volunteers [38]. Therefore, breath-hold scans are not recommended for clinical renal ASL **[R7.3]** and scans under free breathing are preferred **[R7.4]**. Respiratory triggering methods have been widely used in renal ASL [16, 18, 43, 44, 58, 73] (among others) and can reduce motion artefacts in free breathing scans but at the expense of scan time **[R7.5]**. Respiratory coaching, where subjects are instructed to breathe during the periods when no data are being acquired (i.e. time gaps between acquisition of control and label data), have also been used to reduce motion artefacts [55, 57, 62, 74]. External hardware (e.g. bellows) can be used to monitor patient compliance. However, the success of this approach depends on the degree of cooperation of the subject [74], which can limit the applicability of this technique in routine clinical scans.

Even though the degree to which fat suppression is required varies depending on the chosen type of image readout, in general, we recommend the use of fat suppression in renal ASL to avoid artefacts caused by the displacement of MRI signal from fatty tissue **[R7.6]**. This recommendation is particularly important for readout schemes with a low phase-encoding bandwidth, such as the aforementioned recommended EPI readout.

### Data preprocessing

Preprocessing methods retrospectively improve data quality prior to perfusion quantification. Advantages of these methods are that they typically do not involve extra patient preparation or an increase in scan time, but in some cases require local expertise and/or can be time-consuming.

The standard preprocessing operation in ASL data is voxelwise signal averaging of the multiple control-label measurements acquired at a single TI (in PASL) or PLD (in PCASL). This addresses the inherently low SNR of ASL data and provides a degree of motion robustness provided a significant fraction of the ASL data is acquired with the kidneys at a consistent position. Other preprocessing methods in renal ASL aim to remove or downweight outlier measurements or correct corrupted individual perfusion weighted-images, most frequently caused by subtraction errors due to kidney motion (see Figure S2 in the supplementary material). Outlier rejection methods, including retrospective sorting of renal ASL data, have relied on manual [44, 56, 62] or automatic [18, 58] approaches, including using data from external sensors such as respiratory bellows [55, 63]. Outlier rejection methods have been shown beneficial to improve the data quality and, therefore, are recommended for renal ASL **[R8.2]**.

Another class of preprocessing methods are motion correction techniques typically based on image registration, which can be used in conjunction with outlier rejection. These methods are widely used in renal ASL [11, 18, 63, 75, 76] (among others) to realign the kidneys and ensure their position is consistent throughout the ASL time series and, therefore, reduce subtraction errors. They have been shown to reduce image artefacts, reduce partial volume effects between cortex and medulla, and improve the SNR of the perfusion-weighted signal and the repeatability of perfusion measurements. As such, retrospective image registration is highly recommended for renal ASL **[R8.1]**. Note that the kidneys should be registered separately if using rigid/affine transformations as they move independently [77, 78]. In addition, these methods should be used to not only align ASL control and label images but also any calibration scans [18, 21], such as M_0_ data (essential for quantification) and optional T_1_ data (acquisition of which is currently not recommended as part of the default renal ASL protocol). Extra care should be taken when aligning the ASL data to the calibration data (particularly if BGS is used) due to differences in image contrast which can reduce the accuracy of registration algorithms. Smoothing the M_0_ data may reduce the impact of misregistration on perfusion quantification [54, 79]. Methods to address this issue directly have been proposed (e.g. [11].) but since they require further validation and/or changes to the acquisition scheme, they are currently not recommended.

### Quantification

Quantifying perfusion images in physiologic units is an important step in the processing of ASL. Accurate quantification enables comparison of values across scans, subjects, MR scanners, and even non MRI blood flow measurement techniques. Ideally, quantification also removes sensitivity to technical and physiologic factors not directly related to perfusion. Methods for quantification of renal perfusion draw heavily on the wider experience with ASL in the brain [27, 54, 80, 81]. These methods relate the control-label signal differences calculated above to a model for the signal dependence on tissue perfusion. As with most models of measurements in biological systems, there is a tradeoff between measurement feasibility and the greater modelling accuracy provided by more complex models. Concurrently, efforts in improving accuracy may be hampered by inherent random variability in the data which in the presence of complex models may yield noisier parameter estimates. Here, we recommend quantification using a simpler model for the ASL signal that requires very few additional measurements. This model neglects MRI signal relaxation differences between blood and tissue so it is referred to as a single-compartment model. An advantage of this model, and our recommended single delay acquisition approach **[R9.2]**, is that the tissue perfusion at each image voxel can be calculated directly by an equation, without iterative fitting. The equation to calculate renal perfusion (commonly referred to as renal blood flow, RBF), for PCASL is:

1 $$ {\text{RBF}} = \frac{{6000 \cdot \lambda \cdot \left( {{\text{SI}}_{{{\text{control}}}} - {\text{SI}}_{{{\text{label}}}} } \right) \cdot {\exp}\left( {{\text{PLD}}/T_{{1,{\text{blood}}}} } \right)}}{{2 \cdot \alpha \cdot T_{{\text{1,blood}}} \cdot {\text{SI}}_{{{\text{PD}}}} \cdot \left( {1 - {\exp}\left( { - \tau /T_{{1,{\text{blood}}}} } \right)} \right)}}\;\left[ {{\text{mL}}/{1}00\;{\text{g}}/{\min}} \right], $$

while for FAIR with QUIPSSII type saturation to shape the bolus the corresponding equation is:

2 $$ {\text{RBF}} = \frac{{6000 \cdot \lambda \cdot \left( {{\text{SI}}_{{{\text{control}}}} { } - {\text{SI}}_{{{\text{label}}}} } \right) \cdot {\exp}\left( {{\text{TI}}/{\text{T}}_{{\text{1,blood}}} } \right)}}{{2 \cdot \alpha \cdot {\text{TI}}_{1} \cdot {\text{SI}}_{{{\text{PD}}}} }}\;\;\left[ {{\text{mL}}/{1}00\;{\text{g}}/{\min}} \right] $$

Note that RBF is closely related to renal plasma flow (RPF), RPF = ( 1− Hct)*RBF, the latter typically estimated in Para-aminohippurate (PAH) clearance studies. In these equations: $T_{{\text{1,blood}}}$ is the MR longitudinal relaxation time of arterial blood, assumed to be constant across subjects and recommended to be 1.65 s at 3 T **[R9.5]** and 1.48 s at 1.5 T **[R9.6]** acquisitions, $SI$ with the respective subscripts represents the signal of the control, label, and proton density (PD) images (see below), $\alpha$ is the assumed labelling efficiency **[R9.7, R9.8]**, and $\lambda$ is the assumed constant value for the tissue–blood partition coefficient, defined as the grams of water per gram of tissue divided by the grams of water per mL of blood. Since a reliable reference for the partition coefficient value in kidney was not known to this group, we recommend the use of a value of 0.9 mL/g **[R9.4]**, the average value for brain tissue. Indeed, literature on water content in the renal cortex suggests the value of 0.9 mL/g to be a good approximation for the partition coefficient value in kidney [82–84]. Since this is a constant factor across the image, perfusion values calculated with this assumption could be readily corrected when a more accurate value of $\lambda$ is known. $PLD$ and $\tau$ are the postlabelling delay and labelling duration of the PCASL acquisition, while $TI$ and ${\text{TI}}_{{1}}$ are the inversion time and bolus duration of the QUIPSSII/Q2TIPS type acquisition. If multiple slices are acquired with an echo-planar acquisition, ${\text{PLD}}$ or ${\text{TI}}$ will be longer for later acquired slices. These values should be determined for a given slice by incrementing the ${\text{PLD}}$ or ${\text{TI}}$ for the first slice with the $\left( {{\text{slice number}} - {1}} \right)\; \times \;{\text{slice acquisition interval}}$. The efficiency, $\alpha$, should be multiplied by an additional factor of 0.93 for each BGS inversion pulse added **[R9.9]** [69]. All recommended values are provided in Table 2. Scaling the perfusion-weighted signal into quantitative units (typically mL/100 g/min) requires estimation of the equilibrium magnetization in fully relaxed arterial blood. This is typically estimated from a separately acquired PD image and the aforementioned tissue–blood partition coefficient. As such, we consider acquisition of a separate PD image (also referred to as *M*_0_ image) a mandatory step for ASL quantification **[R.9.1]**. The PD image should be acquired without labelling or BGS and using a similar readout and acquisition parameters, with the exception that a long TR should be used. If this image is acquired without waiting for a sufficiently (> 5 s) long recovery time (TR), the ${\text{SI}}_{{{\text{PD}}}}$ should be corrected for incomplete relaxation using the equation:

3 $$ {\text{SI}}_{{\text{PD,corr}}} = \frac{{{\text{SI}}_{{{\text{PD}}}} }}{{1 - {\exp}\left( { - {\text{TR/T1,}}\;{\text{tissue}}} \right)}}, $$

where $T_{{\text{1,tissue}}}$ is an estimate of the kidney T_1_.

For quantitative assessment of renal perfusion, we recommend region of interest (ROI) analysis to estimate an average perfusion value from the quantitative perfusion map **[R9.10]**. Renal cortex perfusion is most reliable, because its flow is high and the cortex tends to be more distant from the collecting system and major arteries that can cause artifacts on ASL. ROIs should be drawn manually on an anatomic image to avoid biases, either the PD image or a spatially registered higher resolution anatomic image **[R9.11]**. Semi-automatic methods may be used if local expertise is available (e.g. using T_1_ maps) but require extensive validation. ROIs should be adjusted to avoid hyperintense signals on the perfusion image as they likely represent vessels. Medullary perfusion is difficult to measure reliably [56] **[R10.2**] because of its lower perfusion (and, therefore, lower signal) and close proximity to cortex which makes it susceptible to partial volume contamination. The kinetics of labelled water are also uncertain because arterial water is divided between filtrate and smaller arterioles. This water may also exchange with surrounding tissue before penetrating deep into the medulla. In spite of the above mentioned issues, if an estimate of medullary perfusion is of interest it would be critical to carefully select ROIs to include sufficient medullary voxels to improve statistical power while attempting to minimize partial volume effects, as well as potentially increasing scan time (averaging) to boost SNR.

The recommended single-compartment model and quantification method neglects a range of effects that the panel considered too complicated to measure in a typical clinical research or clinical study at the current experience level with renal ASL. Importantly, we neglect the difference in T_1_ between blood and renal tissue. This is justifiable because the difference is not very large and the assumption of equal T_1_ greatly simplifies the equation. Though quantification of T_1_ is also an important method for renal characterization with ASL, including the subject or even voxel-specific value of T_1_ would also require measurement of the time after labelling when blood water exits the microvasculature and enters the tissue. Measurement of arrival kinetics by multiple delay or TI acquisition and quantification by fitting a two-compartment model with separate transit time and tissue T_1_ measurements is a viable alternative to the single-compartment approach but requires more complex acquisition/analysis methods and, therefore, is currently not recommended as the default renal ASL approach **[R9.3]**. We also assume ranges of transit times from the labelling region to the tissue that are sufficient for the equations above to be valid. T_1_ of blood can vary across subjects and time, especially with differences in haematocrit [85], and kidney water content may change with pathology, affecting the quantification through T_1_ [44] and the tissue–blood partition coefficient. As such, care should be taken in the interpretation of RBF measurements, or when comparing longitudinal RBF measurements, in conditions where the haematocrit is likely to change (e.g. anemia). The efficiency of labelling may also vary across subjects and time, especially if there are large changes in aorta and renal artery blood flow velocities.

Though we have recommended the use of saturation to define the bolus duration in pulsed labelling [18, 86], many studies of renal perfusion with pulsed ASL have not included this saturation. Quantification is typically performed by replacing TI_1_ with TI in the RBF equation [63, 87]. This approach maximizes signal but will underestimate flow by a transit time-dependent factor.

Several imaging studies have shown that a lower cortical thickness is associated with reduced renal function as measured by eGFR [88–91]. In the case of advanced disease, the reduction in cortical thickness can be severe enough such that it approaches the typical dimensions of the ASL voxels, significantly reducing the number of pure cortex voxels from which cortical RBF can be estimated. The mixing of perfusion signal from the cortex with medullary signal may bias cortical RBF estimates to lower values (i.e. potentially causing an apparent reduction of cortical perfusion). Partial volume correction methods can be helpful in addressing this issue. However, the current lack of thoroughly tested (especially in renal ASL) and user-friendly partial volume correction methods hinders their widespread use. Therefore, cortical RBF results from ASL should be interpreted with caution in cases where cortical thinning is evident [92].

### Data analysis/reporting

A recent systematic review of the renal ASL literature highlighted wide variation in how renal ASL data were reported making it difficult to compare and evaluate studies [8]. A strong agreement among the panel (81–100%) was found for reporting the general and ASL-specific parameters summarized in Table 4.

**Table 4:** Minimum set of parameters to be reported in ASL studies

General MR parameters	Scanner manufacturer/model, receive coil type, pixel bandwidth, fat suppression, field of view, magnetic field strength, flip angle, image orientation, in-plane resolution, number of slices, parallel imaging (technique and acceleration factor), partial Fourier, physiological triggering/gating, readout pulse sequence type, slice gap, slice ordering, slice thickness, echo time, repetition time
ASL-specific parameters	Background suppression, Inflow time(s)/post-labeling delay(s), labeling duration, labeling type, number of averages, quantification model

There was also consensus that a minimum of mean and standard deviation of cortical renal ASL perfusion values should be reported at the subject (i.e. ROI analysis) or group level. The median should also be considered in the presence of skewed RBF distributions. Values for the right and left kidney should be reported separately **[R10.1]**. At the time of writing, the panel could not yet recommend reporting medullary perfusion values by ASL due to poor reproducibility but noted that this was an area of active research **[R10.2]**.

### Transplants

This section describes specific aspects that should be taken into account for ASL of transplant kidneys that are different from the recommendations described above for native kidneys. There is a promising translational potential for functional renal MRI in the transplanted kidney given that 28% of deceased donor and 15% of living donor kidneys will undergo chronic rejection within 5 years [93] and biopsy monitoring is invasive and with limitations such as sampling bias. Specifically, functional renal MRI has shown promising results for detection of acute tubular necrosis (ATN) [94] and longitudinal decline in renal perfusion within the first 2 years of transplant [73].

**Fig. 7:**
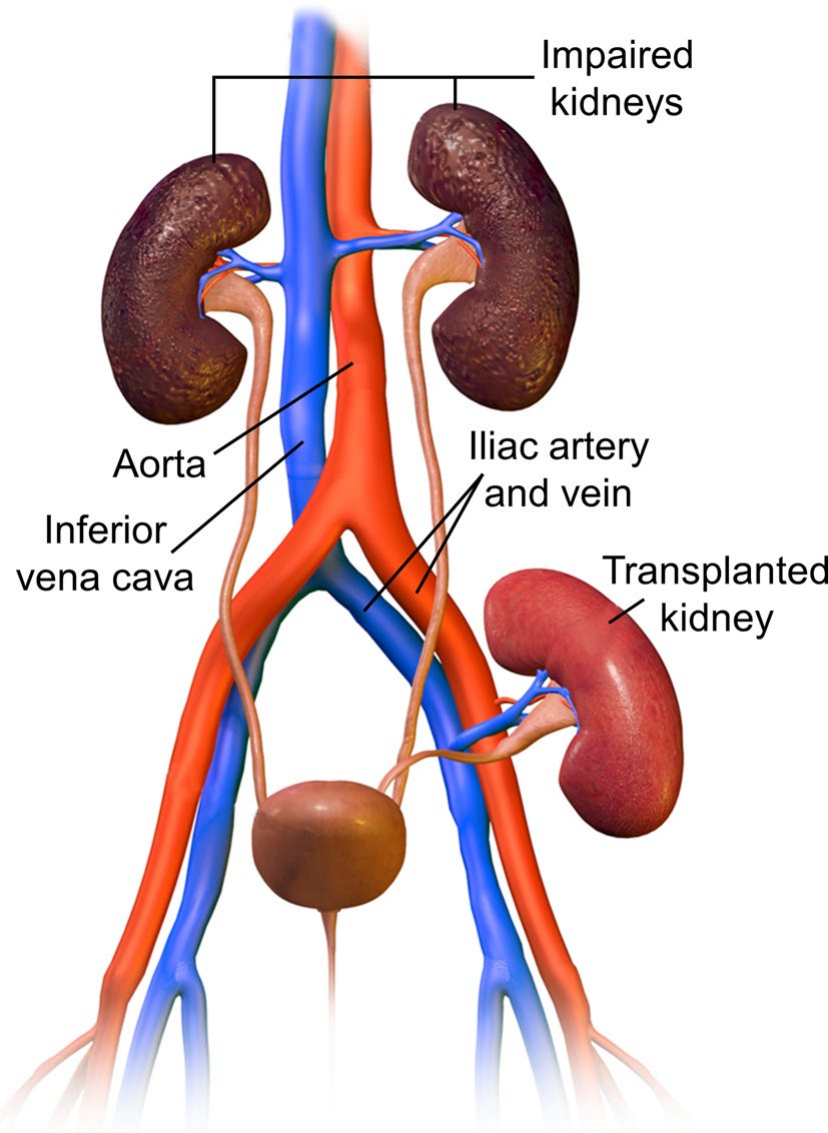
Renal transplantation and vascular anatomy. Adapted from https://commons.wikimedia.org/wiki/File:Kidney_Transplant.png. [Accessed 21 Oct 2019] under the Creative Commons Attribution-Share Alike 4.0 International license

Most commonly, the transplanted kidney is placed in the lower abdomen near the (right) iliac fossa and the renal artery and vein are connected to the external iliac artery and vein, respectively. The kidney body orientation varies and is atypical when transplanted to the lower abdomen making the transplant setting unique for renal ASL. In the native kidneys, the renal arteries commonly follow a horizontal or slightly downward trajectory (i.e. flow direction is medial-to-lateral). The frequent oblique orientation of the artery for the renal allograft after transplantation is such that the direction of flow in this vessel is both medial-to-lateral and inferior-to-superior (i.e. caudo–craneal) (see Fig. 7). The direction of flow in the iliac artery remains craneo–caudal after transplantation. However, the baseline flow from the iliac artery likely changes the absolute perfusion of the transplanted kidney compared to the native kidney, biasing absolute perfusion towards lower values [73]. The lower abdomen and transplanted kidney are much less influenced by breathing motion compared to native kidneys, making respiratory gating/triggering less important for ASL in the transplant setting.

**Fig. 8:**
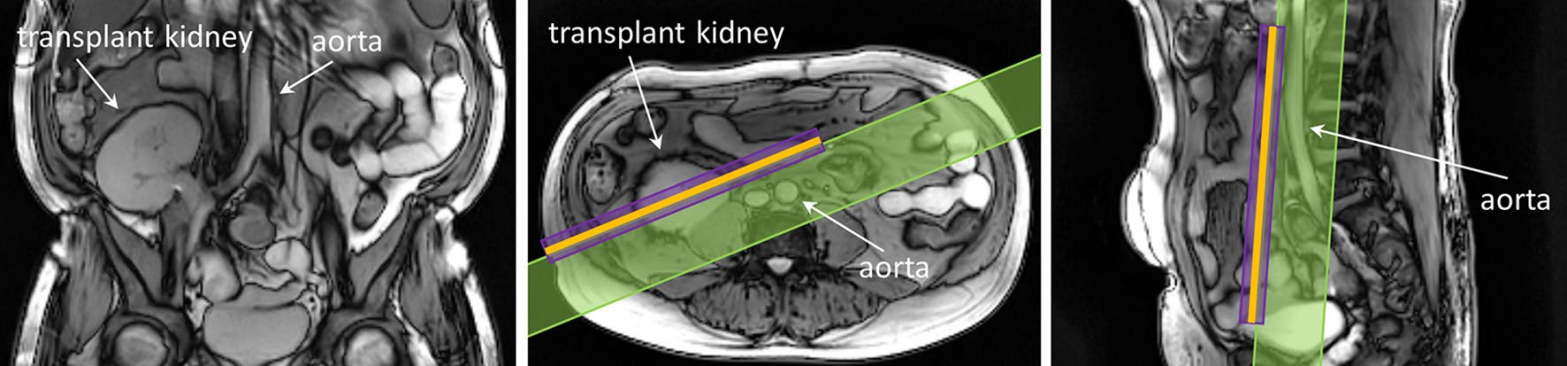
Positioning of the FAIR selective inversion slab (in purple), imaging slice (in orange) and QUIPSSII type saturation slab (in green) is shown on anatomical localizers in the coronal, axial and sagittal orientations for a transplant kidney. Planning of the FAIR ASL scan for transplant kidneys can be challenging due to the necessity of avoiding the inclusion of the aortic and iliac feeding arteries within the selective inversion slab

However, anatomical orientation of the kidney transplant can vary between patients. To keep the same image orientation with respect to the inflowing blood direction as used for native kidneys, the plane of the image acquisition should be oriented along the long axis of the kidney body. For FAIR ASL-based methods, it can be challenging to plan the positioning of the imaging and selective inversion planes along the long axis of the transplanted kidney, while at the same time avoiding the abdominal aortic and iliac feeding arteries (see Fig. 8). Although FAIR ASL has been shown to be feasible and repeatable using an oblique sagittal orientation [76], PCASL and related methods that provide greater flexibility in positioning of the imaging and labelling planes may be preferable in the transplant setting. It is recommended to plan the PCASL labelling plane perpendicular to the abdominal aorta, just above the bifurcation into the iliac arteries (see Fig. 7). This is a clear landmark to use for planning, independent of a left- or right-sided transplant location, and ensures that the labelling plane does not coincide with the kidney transplant. It is worth noting that even though blood flow velocity is lower at this location [95], the recommended PCASL parameters should still enable efficient labelling.

Furthermore, readout recommendations for transplanted kidneys are the same as those for native kidneys.

## Limitations

Despite efforts in the inclusion of as broad representation of renal ASL experts as possible, the panel that participated in this consensus formation process was of limited size (*n* = 23), which can be considered a shortcoming of this work. However, it included scientists and clinicians from groups that have had a major impact on the development of renal ASL and its application. The proportion of technically oriented panel members was higher than that of clinically oriented ones. Although the involvement of more clinicians would have been desirable, this is justified given the current state of development of renal ASL. Although most major technical hurdles have been resolved, the use of ASL in the kidney is still confined to a limited number of dedicated research groups.

During the process of consensus formation, panel members were instructed to score the statements considering data available in the renal ASL literature, whenever possible. Another limitation of this work is that experimental data were lacking to provide a definitive answer for certain issues. When no published data were available to support a specific statement or the existing evidence was not strong, members were asked to provide an answer based on their expert opinion. The consensus protocol proposed here can thus be considered a starting point that will likely be modified in the future as more data become available, since the literature in the field is rapidly expanding.

In spite of the growing interest in renal ASL, data on clinical applications are still scarce. Thus, the provided protocol is intended as a tool to promote the clinical translation of the technique to be used mostly in clinical research studies, rather than for direct application in patient care.

Finally, the recommended protocol parameters have been selected to provide robust measurements of cortical renal perfusion, since acquired data on medullary perfusion are not considered to be well validated with current measurement approaches.

## Future directions

We expect future research to address some of the limitations in these recommendations. Lack of direct comparisons of ASL labelling schemes in the kidneys precluded us from recommending a single labelling strategy. More studies on patient populations are required to clarify the utility of ASL for characterizing renal disease and affecting patient management. We recognize that no single standardized protocol will be ideal for all clinical applications but at the very least a wider application of renal ASL will inform where the present recommendations are suboptimal. An example might be cases where renal pathology which causes significant haemodynamic and/or microstructural changes may require more complex acquisitions (e.g. multiple time points for mapping arterial transit times) and/or analyses (e.g. two-compartment modelling to account for differences between blood and tissue *T*_1_).

In parallel to the uptake of renal ASL for clinical studies, we expect future technical developments to continue improving renal ASL measurements. Perfusion measurements in the medulla have only been reported in a small subset of renal ASL studies (e.g. [56, 63, 71]), probably due to technical challenges, as outlined previously in the quantification section, which lead to a lower reproducibility when compared to the cortex. However, recent reports suggest an increasing feasibility of medullary perfusion mapping [15, 16, 96] and clinical need is likely to continue driving developments in this application as alterations in medullary perfusion may be implicated in certain pathologies characterized by renal tubular injury (e.g. AKI). This, and other applications, may be further potentiated by improvements in data processing and analysis pipelines, which we expect in the future to more effectively address remaining common artefacts such as motion, image distortion and/or partial volume effects while also optimizing clinical workflows by enabling efficient visualization and interpretation of perfusion-weighted data and quantitative perfusion maps through streamlined software. ASL is also anticipated to be an important component of multiparametric MRI protocols where the synergistic combination with other non-invasive MRI biomarkers will likely deliver greater diagnostic value [60].

## Conclusions/summary

In summary, this work provides a recommended protocol for renal ASL chosen by consensus by a panel of experts in the field. It aims to provide a starting point to facilitate the use of renal ASL to those lacking experience and to promote the standardization of the acquisition and analysis steps in order to enable data comparison across centers and the establishment of multi-center clinical studies.

As a default protocol we have recommended PCASL or FAIR labelling with a single-slice SE-EPI readout, with background suppression, and a simple but robust quantification model. This should provide renal cortical perfusion images of adequate quality and SNR for most applications. For conditions where extended kidney coverage is desirable, a multislice SE-EPI readout can be employed. 3D readouts are promising but the lack of experience in renal ASL precludes recommendation of their use as the default approach.

With these recommendations we do not intend in any way to slow innovation or development in the field. Moreover, based on the panel discussions, certain issues have been identified as research priorities, including areas where strong evidence would be desirable, as highlighted in the “Future directions” section.

## Electronic supplementary material

Below is the link to the electronic supplementary material.

Supplementary material 1 (TIF 3,121 kb)

Supplementary material 1 (TIF 2,393 kb)

Supplementary material 1 (DOCX 470 kb)
