# Supplementary figures and images for: Consensus-based technical recommendations for clinical translation of renal ASL MRI
Source: MAGMA. 2019 Dec 12;33(1):141–61. doi: 10.1007/s10334-019-00800-z (PMC7021752; doi:10.1007/s10334-019-00800-z)

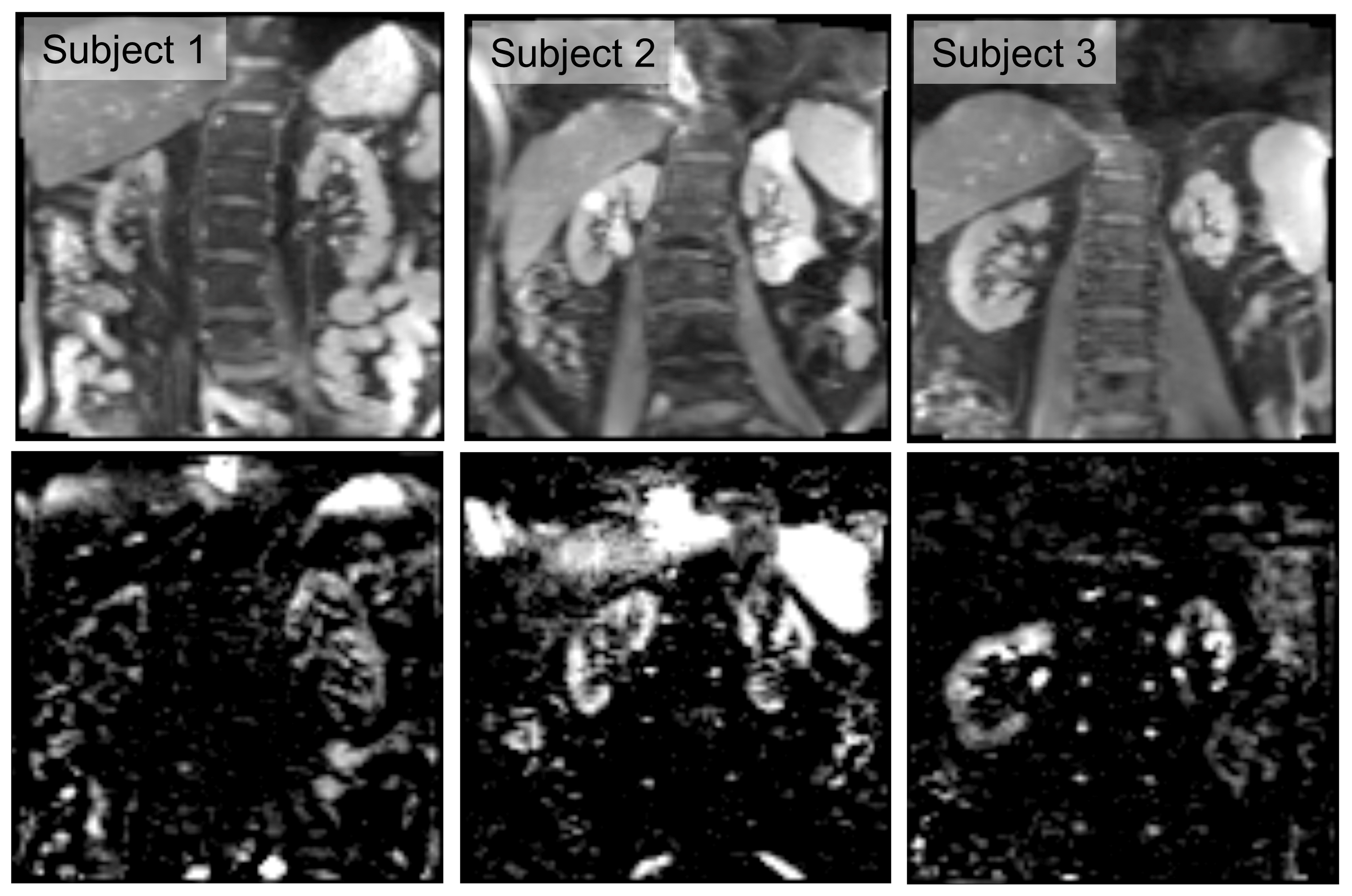

Supplement: Supplementary file 1 — Supplementary material 1 (TIF 3,121 kb) [file 10334_2019_800_MOESM1_ESM.tif]

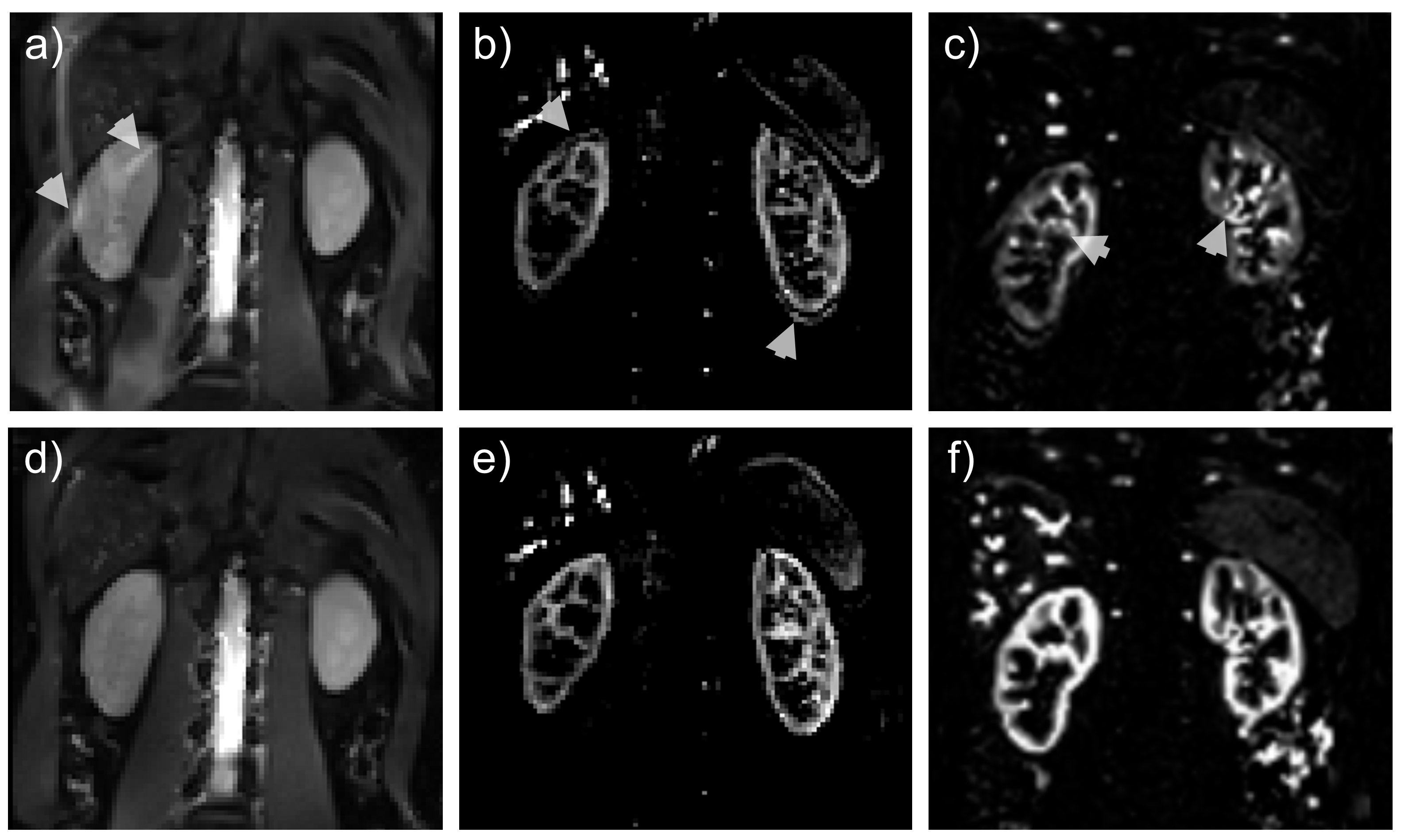

Supplement: Supplementary file 2 — Supplementary material 1 (TIF 2,393 kb) [file 10334_2019_800_MOESM2_ESM.tif]
