# Supplementary material for: Consensus-based technical recommendations for clinical translation of renal ASL MRI
Source: MAGMA. 2019 Dec 12;33(1):141–61. doi: 10.1007/s10334-019-00800-z (PMC7021752; doi:10.1007/s10334-019-00800-z)
Supplement: Supplementary file 3 — Supplementary material 1 (DOCX 470 kb) [file 10334_2019_800_MOESM3_ESM.docx]

Consensus-based technical recommendations for clinical translation of renal ASL MRI

*Supplementary information*

Fabio Nery^1^, PhD, Charlotte E. Buchanan^2^, PhD, Anita A. Harteveld^3^, PhD, Aghogho Odudu^4^, PhD, Octavia Bane^5^, PhD, Eleanor F. Cox^2^, PhD, Katja Derlin^6^, MD, H. Michael Gach*^7^*, PhD, Xavier Golay^8^, PhD, Marcel Gutberlet^6^, PhD, Christoffer Laustsen^9^, PhD, Alexandra Ljimani^10^, MD, Ananth J. Madhuranthakam^11^, PhD, Ivan Pedrosa^11^, MD, PhD, Pottumarthi V. Prasad^12^, PhD, Philip M. Robson^5^, PhD, Kanishka Sharma^13^, PhD, Steven Sourbron^13^, PhD, Manuel Taso^14^, PhD, David L. Thomas^8^, PhD, Danny J.J. Wang^15^, PhD, Jeff L. Zhang^16^, PhD, David C. Alsop^14^, PhD, Sean B. Fain^17^, PhD, Susan T. Francis^2^, PhD, María A. Fernández‐Seara^18^*, PhD

*^1^Developmental Imaging and Biophysics Section, UCL Great Ormond Street Institute of Child Health, London, United Kingdom.*

*^2^Sir Peter Mansfield Imaging Centre, School of Physics and Astronomy, University of Nottingham, Nottingham, United Kingdom.*

*^3^Department of Radiology, University Medical Center Utrecht, Utrecht University, Utrecht, The Netherlands.*

*^4^Division of Cardiovascular Sciences, School of Medical Sciences, Faculty of Biology, Medicine and Health, University of Manchester, Manchester, United Kingdom.*

*^5^Translational and Molecular Imaging Institute and Department of Radiology, Icahn School of Medicine at Mount Sinai, New York, NY, USA.*

*^6^Department of Radiology, Hannover Medical School, Hannover, Germany.*

*^7^Departments of Radiation Oncology, Radiology, and Biomedical Engineering, Washington University in St. Louis, St. Louis, MO, USA.*

*^8^Department of Brain Repair and Rehabilitation, UCL Queen Square Institute of Neurology, University College London, London, United Kingdom.*

*^9^MR Research Centre, Department of Clinical Medicine, Aarhus University, Aarhus, Denmark.*

*^10^Department of Diagnostic and Interventional Radiology, Medical Faculty, Heinrich Heine University Düsseldorf, Düsseldorf, Germany.*

*^11^Department of Radiology and Advanced Imaging Research Center, UT Southwestern Medical Center, Dallas, TX, USA.*

*^12^Department of Radiology, Center for Advanced Imaging, NorthShore University Health System, Evanston, IL, USA.*

*^13^Imaging Biomarkers Group, Department of Biomedical Imaging Sciences, University of Leeds, UK.*

*^14^Division of MRI Research, Department of Radiology, Beth Israel Deaconess Medical Center and Harvard Medical School, Boston, MA, USA.*

*^15^Stevens Neuroimaging and Informatics Institute, University of Southern California, CA, USA.*

*^16^A.A. Martinos Center for Biomedical Imaging, Massachusetts General Hospital, Harvard Medical School.*

*^17^University of Wisconsin – Madison, Departments of Medical Physics, Radiology, and Biomedical Engineering.*

*^18^Department of Radiology, Clínica Universidad de Navarra, Pamplona, Spain.*

***Corresponding author:** María A. Fernández‐Seara, Department of Radiology, Clínica Universidad de Navarra, Pamplona, Spain. Email: mfseara@unav.es. Tel.: +34 948255400. Fax: 948296500

Supplementary figures


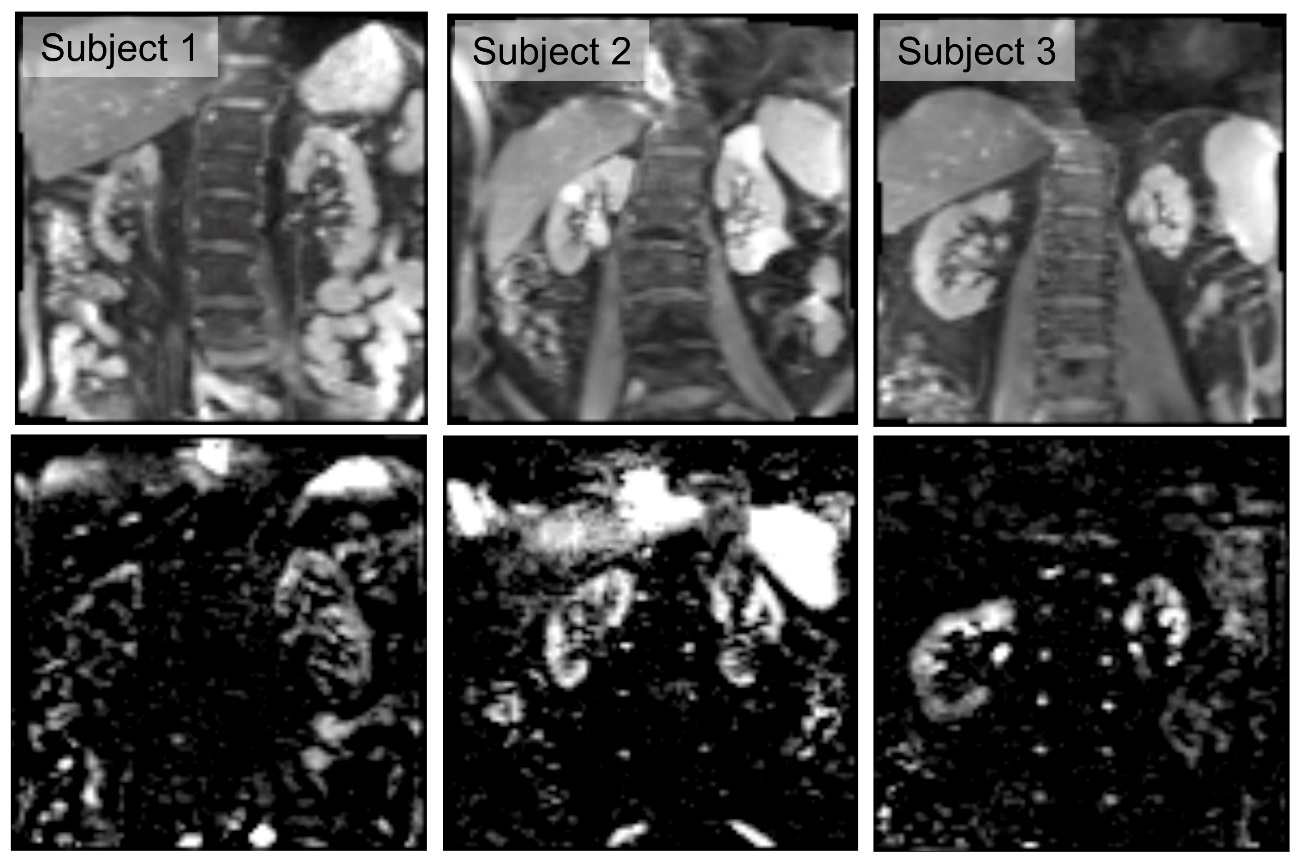


Figure S1 Examples of renal ASL data in three patients with chronic kidney disease (stage 3). Top row: M_0_ images; Bottom row: ASL mean perfusion-weighted images.


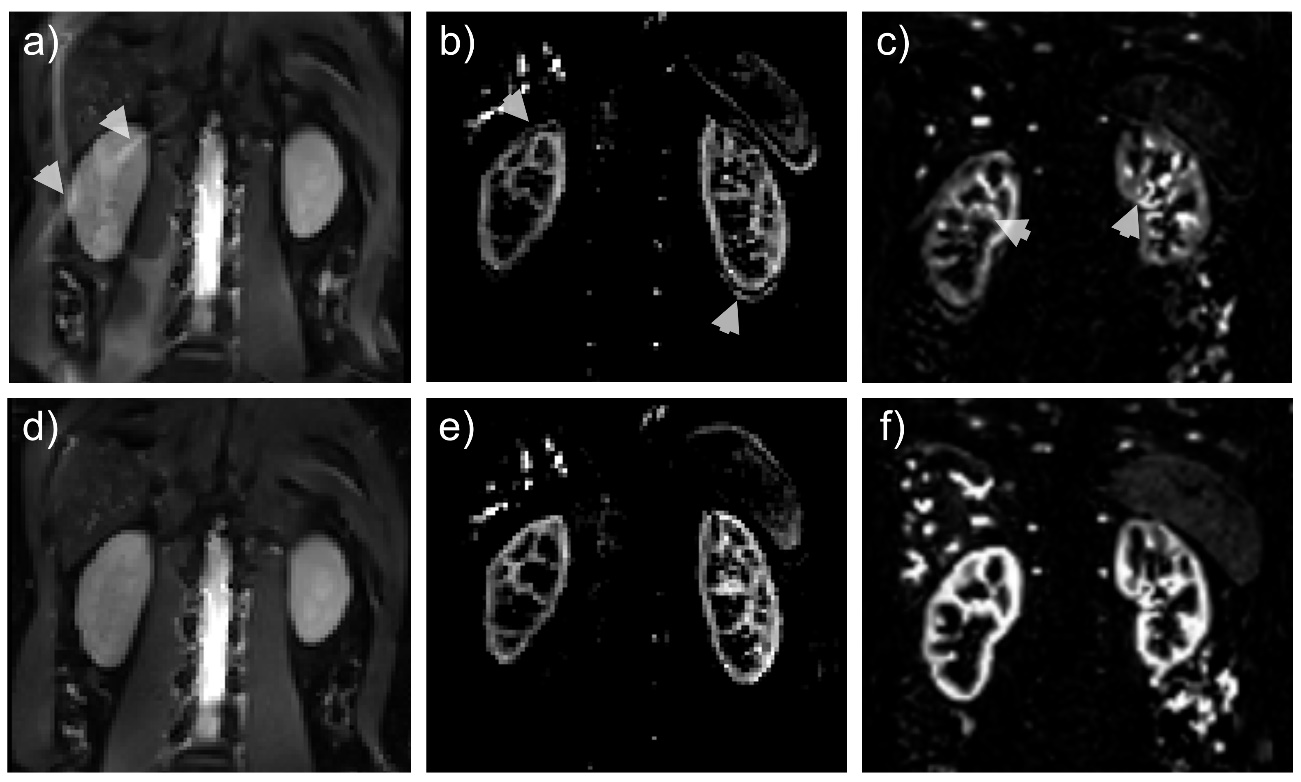


Figure S2 Examples of possible artefacts in renal ASL data. The top row shows the images with artefacts and the bottom row shows “artefact-free” data. Artefacts shown include a) chemical shift (show in M_0_ data); b) motion artefacts due to subtraction of control and label data where the kidneys are in inconsistent positions and c) arterial transit time artefacts. Note the arrowheads pointing to the image artefacts. Both b) and c) and the corresponding “artefact-free” images (e) and f)) are ASL difference images.

Initial Survey (S0)

The goal with this survey was to obtain data to assist the panel co-chairs in drafting an initial set of consensus statements considering feedback from the entire panel. The questions in this survey allow a broad range of possible replies which were not standardized unlike in later surveys. Therefore, all possible replies to each question are reproduced here (marked bellow with a bullet symbol (•)).

# 1. Patient preparation

## 1.1. Fasting before scanning? [choose a single option]

- Yes (enter duration in “Other” field, in hours)
- No
- No recommendation
- Do not know
- Other…

## 1.2. Restriction on liquids before scanning? [choose a single option]

- Yes (enter duration in “Other” field, in hours)
- No
- No recommendation
- Do not know
- Other…

## 1.3. Any other patient preparation procedure? [choose a single option]

- Yes (elaborate in “Other” field)
- No
- No recommendation
- Do not know
- Other…

# 2. Hardware

## 2.1. Field strength? [choose a single option]

- 1.5T
- 3T
- Both
- No recommendation
- Do not know
- Other…

## 2.2. Transmitter coil? [choose a single option]

- Body coil
- No recommendation
- Do not know
- Other…

## 2.3. Receiver coil? [choose a single option]

- Body array coil
- No recommendation
- Do not know
- Other…

# 3. Labelling strategy

## 3.1. Labelling strategy? [choose a single option]

- PASL: FAIR
- pCASL
- Both (optionally elaborate in “Other” field)
- No recommendation
- Do not know
- Other…

# 4. FAIR labelling parameters

## 4.1. Inversion pulse type? [choose a single option]

- FOCI pulse
- Hyperbolic secant
- No recommendation
- Do not know
- Other…

## 4.2. Positioning of the selective slab? [choose a single option]

- Excluding the feeding arteries
- No recommendation
- Do not know
- Other…

## 4.3. Selective inversion slab thickness? [choose one or more options]

- Imaging slab thickness + [1-5] mm
- Imaging slab thickness + [5-10] mm
- Imaging slab thickness + [10-20] mm
- Imaging slab thickness + [20-40] mm
- No recommendation
- Do not know
- Other…

## 4.4. Non-selective inversion slab thickness? [choose one or more options]

- 300mm
- 400mm
- 500mm
- No recommendation
- Do not know
- Other…

## 4.5. Single or multi-TI acquisitions when using FAIR? [choose a single option]

- Single
- Single (with mandatory separate transit time measurement)
- Multi
- No recommendation
- Do not know
- Other…

## 4.6. Inversion time(s) in single-TI acquisitions? [choose one or more options]

- 0.9s
- 1.0s
- 1.1s
- 1.2s
- 1.3s
- 1.4s
- 1.5s
- 1.6s
- 1.7s
- 1.8s
- 1.9s
- 2.0s
- No recommendation
- Do not know
- Other…

## 4.7. For single-TI acquisitions: approach for controlling the temporal width of the bolus? [choose a single option]

- QUIPSS II
- Q2TIPS
- Any of the above
- No recommendation
- Do not know
- Other…

## 4.8. For single-TI acquisitions: number of ASL pairs? [choose one or more options]

- [1-5]
- [5-10]
- [10-20]
- [20-40]
- >40
- No recommendation
- Do not know
- Other…

## 4.9. Inversion time(s) in multi-TI acquisitions? (write the inversion times in the "Other" field as a vector [TI1, TI2, …, TIn])

- No recommendation
- Do not know
- Other…

## 4.10. For multi-TI acquisitions: number of ASL pairs? [choose one or more options]

- [1-5]
- [5-10]
- [10-20]
- [20-40]
- >40
- No recommendation
- Do not know
- Other…

# 5. PCSL parameters

## 5.1. PCASL variant? [choose a single option]

- Balanced
- Unbalanced
- No recommendation
- Do not know
- Other…

## 5.2. Labelling time? [choose one or more options]

- 1.3s
- 1.4s
- 1.5s
- 1.6s
- 1.7s
- 1.8s
- 1.9s
- 2.0s
- No recommendation
- Do not know
- Other…

## 5.3. Orientation of the labelling plane? [choose a single option]

- Perpendicular to the aorta
- No recommendation
- Do not know
- Other…

## 5.4. Distance from labelling plane to centre of the kidney? [choose a single option]

- 8 cm
- 10 cm
- 12 cm
- No recommendation
- Do not know
- Other…

## 5.5. Pulse shape? [choose a single option]

- Hanning
- No recommendation
- Do not know
- Other…

## 5.6. Pulse duration? [choose a single option]

- 500 μs
- No recommendation
- Do not know
- Other…

## 5.7. Pulse spacing (from the centre of one pulse to the centre of the next)? [choose one or more options]

- As short as possible
- 1000 μs
- 1200 μs
- 1400 μs
- No recommendation
- Do not know
- Other…

## 5.8. Average B1? [choose one or more options]

- 1.4 μT
- 1.5 μT
- 1.6 μT
- 1.7 μT
- 1.8 μT
- No recommendation
- Do not know
- Other…

## 5.9. Average gradient (Gave)? [choose one or more options]

- 0.4 μT/m
- 0.5 μT/m
- 0.6 μT/m
- 0.7 μT/m
- 0.8 μT/m
- 0.9 μT/m
- μT/m
- No recommendation
- Do not know
- Other…

## 5.10. Gmax to Gave Ratio? [choose one or more options]

- 5
- 6
- 7
- 8
- 9
- 10
- No recommendation
- Do not know
- Other…

## 5.11. Single or multi-PLD acquisitions when using PCASL? [choose a single option]

- Single
- Single (with mandatory separate transit time measurement)
- Multi
- No recommendation
- Do not know
- Other…

## 5.12. PLD in single-PLD acquisitions? [choose one or more options]

- 0.9
- 1.0
- 1.1
- 1.2
- 1.3
- 1.4
- 1.5
- 1.6
- 1.7
- 1.8
- 1.9
- 2.0
- No recommendation
- Do not know
- Other…

## 5.13. For single-PLD acquisitions: number of ASL pairs? [choose one or more options]

- [1-5]
- [5-10]
- [10-20]
- [20-40]
- >40
- No recommendation
- Do not know
- Other…

## 5.14. PLDs in multi-PLD acquisitions (write the post-labelling delays in the "Other" field as a vector [PLD1, PLD2, …, PLDn])

- No recommendation
- Do not know
- Other…

## 5.15. For multi-PLD acquisitions, number of ASL pairs per PLD [choose one or more options]

- [1-5]
- [5-10]
- [10-20]
- [20-40]
- >40
- No recommendation
- Do not know
- Other…

# 6. Readout parameters

## 6.1. Readout type? [choose one or more options]

- 2D (single-slice)
- 2D (multi-slice)
- 3D
- No recommendation
- Do not know
- Other…

## 6.2. Number of slices in 2D multi-slice? [choose one or more options]

- [2-4]
- [4-6]
- [6-8]
- [8-10]
- >10
- No recommendation
- Do not know
- Other…

## 6.3. Number of slices in 3D? [choose one or more options]

- [5-10]
- [10-15]
- [15-20]
- >20
- No recommendation
- Do not know
- Other…

## 6.4. Slice gap in 2D multi-slice? [choose a single option]

- [0.1-0.5] mm
- [0.5-1] mm
- [1-2] mm
- >2 mm
- No recommendation
- Do not know
- Other…

## 6.5. Slice orientation? [choose a single option]

- Coronal
- Sagittal
- Transverse
- No recommendation
- Do not know
- Other…

## 6.6. Use oblique acquisitions (e.g. coronal oblique along long axis of the kidneys)? [choose a single option]

- Yes
- No
- No recommendation
- Do not know
- Other…

## 6.7. Slice thickness in 2D single slice? [choose one or more options]

- 3 mm
- 4 mm
- 5 mm
- 6 mm
- 7 mm
- 8 mm
- 9 mm
- No recommendation
- Do not know
- Other…

## 6.8. Slice thickness in 2D multi-slice? [choose one or more options]

- 3 mm
- 4 mm
- 5 mm
- 6 mm
- 7 mm
- 8 mm
- 9 mm
- No recommendation
- Do not know
- Other…

## 6.9. Slice thickness in 3D? [choose one or more options]

- 2 mm
- 3 mm
- 4 mm
- 5 mm
- 6 mm
- 7 mm
- 8 mm
- 9 mm
- No recommendation
- Do not know
- Other…

## 6.10. In-plane resolution? [choose one or more options]

- 2 mm
- 2.5 mm
- 3 mm
- 3.5 mm
- 4 mm
- 4.5 mm
- 5 mm
- No recommendation
- Do not know
- Other…

## 6.11. Partial Fourier phase-encoding? [choose one or more options]

- None
- 60-70%
- 70-80%
- 80-90%
- No recommendation
- Do not know
- Other…

## 6.12. Partial Fourier slice (partition)-encoding in 3D? [choose one or more options]

- None
- 60-70%
- 70-80%
- 80-90%
- No recommendation
- Do not know
- Other…

## 6.13. Use parallel imaging? [choose a single option]

- Yes
- No
- No recommendation
- Do not know
- Other…

## 6.14. Parallel imaging acceleration factor? [choose a single option]

- None
- 2
- 3
- No recommendation
- Do not know
- Other…

## 6.15. TR (including labelling + readout)? [choose one or more options]

- 3s
- 4s
- 5s
- 6s
- 7s
- 8s
- 9s
- No recommendation
- Do not know
- Other…

## 6.16. Readout sequence for single slice 2D? [choose one or more options]

- bSSFP
- SE-EPI
- GRE-EPI
- FLASH/SPGRE/CE-FFE-T1
- No recommendation
- Do not know
- Other…

## 6.17. Readout sequence for multi-slice 2D? [choose one or more options]

- bSSFP
- SE-EPI
- GRE-EPI
- FLASH/SPGRE/CE-FFE-T1
- No recommendation
- Do not know
- Other…

## 6.18. Readout sequence for 3D? [choose one or more options]

- 3D-GRASE
- 3D-RARE
- No recommendation
- Do not know
- Other…

## 6.19. Use segmented 3D sequences? [choose a single option]

- Yes
- No
- No recommendation
- Do not know
- Other…

# 7. Other sequence details

## 7.1. Use presaturation pulses? [choose a single option]

- Yes
- No
- No recommendation
- Do not know
- Other…

## 7.2. Use postsaturation pulses? [choose a single option]

- Yes
- No
- No recommendation
- Do not know
- Other…

## 7.3. Use background suppression? [choose a single option]

- Yes
- No
- No recommendation
- Do not know
- Other…

## 7.4. Breathing scheme? [choose a single option]

- Breath hold
- Free breathing
- Synchronized breathing
- No recommendation
- Do not know
- Other…

## 7.5. Motion compensation? [choose one or more options]

- None
- Respiratory triggering (expiration)
- Respiratory triggering (inspiration)
- Navigators
- No recommendation
- Do not know
- Other…

# 8. Data preprocessing

## 8.1. Motion correction? [choose one or more options]

- None
- Image registration (rigid)
- Image registration (affine)
- Image registration (non-rigid)
- No recommendation
- Do not know
- Other…

## 8.2. Outlier detection and rejection? [choose a single option]

- None
- Yes (visual sorting)
- Yes (automated)
- No recommendation
- Do not know
- Other…

# 9. Quantification

## 9.1. M0 acquisition? [choose a single option]

- Yes
- No
- No recommendation
- Do not know
- Other…

## 9.2. Perform dedicated kidney tissue T1 measurement? [choose a single option]

- Yes
- No
- No recommendation
- Do not know
- Other…

## 9.3. Quantification model? [choose a single option]

- Single compartment/blood T1
- Single compartment/tissue T1
- Two compartments
- No recommendation
- Do not know
- Other…

## 9.4. Blood-tissue partition coefficient? [choose a single option] (enter a single value in the "Other" field (float))

- No recommendation
- Do not know
- Other…

## 9.5. Assumed blood T1 at 3T? [choose a single option] (enter a single value in the "Other" field (float))

- No recommendation
- Do not know
- Other…

## 9.6. Assumed blood T1 at 1.5T? [choose a single option] (enter a single value in the "Other" field (float))

- No recommendation
- Do not know
- Other…

## 9.7. Assumed tissue T1 at 3T? [choose a single option] (enter a single value in the "Other" field (float))

- No recommendation
- Do not know
- Other…

## 9.8. Assumed tissue T1 at 1.5T? [choose a single option] (enter a single value in the "Other" field (float))

- No recommendation
- Do not know
- Other…

## 9.9. Labelling efficiency PASL? [choose a single option] (enter a single value in the "Other" field (float))

- No recommendation
- Do not know
- Other…

## 9.10. Labelling efficiency PCASL? [choose a single option] (enter a single value in the "Other" field (float))

- No recommendation
- Do not know
- Other…

## 9.11. Perform a separate arrival time measurement? [choose a single option]

- Yes
- No
- No recommendation
- Do not know
- Other…

## 9.12. Region of interest selection method? [choose a single option]

- Manual
- Semi-automatic (please elaborate in “Other” field)
- Automatic (please elaborate in “Other” field)
- No recommendation
- Do not know
- Other…

## 9.13. Dataset for region of interest selection? [choose one or more options]

- ASL perfusion weighted data (difference signal)
- ASL M0 scan
- Separately acquired anatomical scan (e.g. localizer)
- No recommendation
- Do not know
- Other…

# 10. Bonus questions

## 10.1. If we want to include a set of recommendations for transplant studies, we would need to fill a separate questionnaire. Do you think we should include recommendations for transplant studies in this paper?

- Yes (optionally elaborate in “Other” field)
- No (optionally elaborate in “Other” field)
- No recommendation
- Do not know
- Other…

Consensus Statements Survey 1 (S1)

For all statements in this survey, respondents were allowed to choose one of the following options

- 1 - Strongly Disagree
- 2 - Disagree
- 3 - Neutral
- 4 - Agree
- 5 - Strongly Agree

Additionally, a free text field was provided for each statement for any additional comments.

# 1. Patient preparation

## 1.1. Fasting is not required before scanning.

## 1.2. Restriction on liquids intake is not required before scanning.

# 2. Hardware

## 2.1. Both 1.5T and 3T are adequate field strengths.

## 2.2. The body coil should be used as transmitter coil.

## 2.3. Body phased-array coils should be used as receive coils.

# 3. Labelling strategy

## 3.1. Both PASL:FAIR and PCASL are adequate labelling strategies.

## 3.2. Single time-point acquisitions are recommended for simplicity of acquisition and data analysis.

## 3.3. Multiple time-point acquisitions require a longer acquisition time and more complicated processing. However, they can provide measurements of RBF and ATT that can be useful if delayed arrival time is suspected in a clinical population.

# 4. FAIR labelling parameters

## 4.1. A FOCI pulse should be used for the selective inversion to optimize the inversion slice profile.

## 4.2. The selective slab should be carefully positioned, excluding the aorta.

## 4.3. The selective inversion slab thickness should equal the imaging slab thickness + [10-20] mm.

## 4.4. In single-TI acquisitions, an inversion time of 1.8-2.0 s is recommended.

## 4.5. In single-TI acquisitions, an approach for controlling the temporal width of the bolus (QUIPSS II or Q2TIPS) must be used to quantify RBF.

## 4.6. A bolus duration (TI1) of 1.0-1.2 s is recommended.

## 4.7. In single-TI acquisitions, 20-30 ASL pairs are recommended.

# 5. PCASL parameters

## 5.1. An unbalanced version of PCASL is preferred due to its lower sensitivity to off-resonance effects.

## 5.2. A labelling time of 1.6-1.8s is recommended.

## 5.3. The labelling plane should be oriented approximately perpendicular to the aorta.

## 5.4. The labelling plane should be positioned at approximately 10 cm from the centre of the kidney, in the superior direction.

## 5.5. Hanning RF pulses are recommended.

## 5.6. An RF pulse duration of 500 μs is recommended.

## 5.7. Pulse spacing (from the centre of one pulse to the centre of the next) of 1000 μs or shorter is recommended.

## 5.8. Average B1 of 1.6 μT is recommended.

## 5.9. Average gradient (Gave) of 0.4-0.6 mT/m is recommended.

## 5.10. Gmax to Gave Ratio of 6-7 is recommended.

## 5.11. In single PLD acquisitions a PLD of 1.2-1.5 s is recommended.

## 5.12. In single-PLD acquisitions, 20-30 ASL pairs are recommended.

# 6. Readout

## 6.1. 3D acquisition schemes (3D-RARE and 3D-GRASE) are recommended if full kidney coverage is necessary.

## 6.2. 2D acquisitions schemes are a viable alternative for reduced coverage.

## 6.3. In 3D acquisitions, the number of slices should be sufficient to cover the entire kidneys.

## 6.4. In 2D multi-slice acquisitions, 6 slices achieve a good compromise between FOV and slice-spacing.

## 6.5. Coronal oblique slices (along the major axis of the kidneys) are preferable for renal ASL.

## 6.6. The recommended slice thickness in 2D acquisitions is 4-8mm.

## 6.7. The recommended slice thickness in 3D acquisitions is 3-6mm.

## 6.8. The recommended in-plane resolution is 2-4mm.

## 6.9. Undersampling methods, such as partial Fourier and parallel imaging at moderate acceleration factors (up to R=2) may be used

## 6.10. The recommended TR (including labelling + readout) is 4-6 seconds.

## 6.11. bSSFP readout preferable for single-slice protocols.

## 6.12. SE-EPI readout preferable for multi-slice protocols. bSSFP are a viable alternative but reduced coverage.

# 7. Other sequence details

## 7.1. Pre and post-saturation pulses are recommended.

## 7.2. Background-suppression is recommended for renal ASL when using 3D readouts.

## 7.3. Breath-hold scans are not recommended for clinical renal ASL.

## 7.4. Renal ASL scans should be performed under free breathing.

## 7.5. Respiratory triggering or navigators are preferable provided increased and/or unpredictable scan time are acceptable.

# 8. Data preprocessing

## 8.1. Retrospective image registration is highly recommended for renal ASL.

## 8.2. Outlier rejection is recommended for renal ASL.

# 9. Quantification

## 9.1. M0 acquisition is mandatory.

## 9.2. Using a single-compartment model with assumed blood T1 for quantification is recommended for robustness and simplicity.

## 9.3. A two-compartment model with separate transit time and tissue T1 measurements is a viable alternative to the single-compartment approach but requires more complex acquisition/analysis methods and therefore is not recommended as the default renal ASL approach.

## 9.4. Blood-brain partition coefficient = 0.8.

## 9.5. Assumed blood T1 at 3T = 1.65s [Zhang2013, 10.1002/mrm.24550].

## 9.6. Assumed blood T1 at 1.5T = 1.48s [Zhang2013, 10.1002/mrm.24550].

## 9.7. Assumed tissue T1 at 3T = 1.15s [deBazelaire2004, 10.1148/radiol.2303021331].

## 9.8. Assumed tissue T1 at 1.5T = 1s [deBazelaire2004, 10.1148/radiol.2303021331].

## 9.9. Labelling efficiency PASL = 95% (neglecting background suppression loss).

## 9.10. Labelling efficiency PCASL = 85% (neglecting background suppression loss).

## 9.11. When background suppression is used, the labelling efficiency needs to be adjusted based on the number of background suppression pulses.

## 9.12. Regions of interest selection should be performed manually as the default approach. Semi-automatic methods may be used if local expertise is available (e.g. using T1 maps) but require extensive validation.

## 9.13. Region of interest selection should be performed based on the ASL M0 image or a separately acquired structural dataset.

Consensus Statements Survey 2 (S2)

Consensus had been reached for several statements by the time this survey (S2) was shared to the panel for scoring (i.e. as a result of the first consensus survey (S1)). They are included here for reference and have been identified by a checkmark symbol (✓).

For the majority^†^ of the remaining statements in this survey still open for scoring (numbered), respondents were allowed to choose one of the following options:

- I agree
- I disagree
- I have insufficient experience to make a recommendation

Additionally, a free text field was provided for each section for any additional comments.

^†^Exceptions were statements 10.1, 10.3 and 10.4. Possible options for each of these are marked bellow with a bullet symbol (•).

# 1. Patient preparation

## 1.1. Diet needs to be controlled before the scan.

## 1.2. Subject should be scanned in a normal hydration status when clinically appropriate.

## 1.3. Subjects are required to follow a controlled and standardized salt intake before the scan.

# 2. Hardware

Consensus has been reached for all statements in this section:

- Both 1.5T and 3T are adequate field strengths.
- The body coil should be used as transmitter coil.
- Body phased-array coils should be used as receive coils.

# 3. Labelling strategy

Consensus has been reached for all statements in this section:

- Both PASL:FAIR and PCASL are adequate labelling strategies.
- Single time-point acquisitions are recommended for simplicity of acquisition and data analysis.
- Multiple time-point acquisitions require a longer acquisition time and more complicated processing. However, they can provide measurements of RBF and ATT that can be useful if delayed arrival time is suspected in a clinical population.

# 4. FAIR labelling parameters

Statements in this section for which consensus has been reached:

- A FOCI pulse should be used for the selective inversion to optimize the inversion slice profile.
- The selective slab should be carefully positioned, excluding the aorta.
- The selective inversion slab thickness should equal the imaging slab thickness + [10-20] mm.
- In single-TI acquisitions, an approach for controlling the temporal width of the bolus (QUIPSS II or Q2TIPS) must be used to quantify RBF.
- A bolus duration (TI1) of 1.0-1.2 s is recommended.

## 4.1. In single-TI acquisitions, an inversion time of 1.8-2.0 s is recommended.

## 4.2. In single-TI acquisitions, a minimum of 20 ASL pairs is recommended.

# 5. pCASL parameters

Statements in this section for which consensus has been reached:

- An unbalanced version of pCASL is preferred due to its lower sensitivity to off-resonance effects.
- The labelling plane should be oriented approximately perpendicular to the aorta.
- Hanning RF pulses are recommended.
- An RF pulse duration of 500 μs is recommended.
- Pulse spacing (from the centre of one pulse to the centre of the next) of 1000 μs or shorter is recommended.
- Average B1 of 1.6 μT is recommended.
- Average gradient (Gave) of 0.4-0.6 mT/m is recommended.
- Gmax to Gave Ratio of 6-7 is recommended.
- In single PLD acquisitions, a PLD of 1.2-1.5 s is recommended.

## 5.1. A labelling time of 1.5-1.8s is recommended.

## 5.2. The labelling plane should be positioned at approximately 8-10 cm from the centre of the kidney, in the superior direction.

## 5.3. In single-PLD acquisitions, a minimum of 20 ASL pairs is recommended.

# 6. Readout

Statements in this section for which consensus has been reached:

- Coronal oblique slices (along the major axis of the kidneys) are preferable for renal ASL.
- The recommended slice thickness in 2D acquisitions is 4-8mm.
- The recommended in-plane resolution is 2-4mm.
- Undersampling methods, such as partial Fourier and parallel imaging at moderate acceleration factors (up to R=2) may be used.
- The recommended TR (including labelling + readout) is 4-6 seconds.

## 6.1. A 2D single-slice acquisition scheme is recommended as the default renal ASL method.

## 6.2. Multislice 2D acquisition schemes are recommended for applications that require extended kidney coverage.

## 6.3. 3D acquisition schemes represent a promising alternative to 2D multislice schemes but are not recommended as the default method for extended kidney coverage due to limited clinical experience with 3D schemes.

## 6.4. Spin-echo EPI is the preferred readout for 2D single-slice acquisitions.

## 6.5. bSSFP and single-shot RARE are adequate alternatives to EPI for 2D single-slice acquisitions.

## 6.6. Spin-echo EPI is the preferred readout for 2D multi-slice acquisitions.

# 7. Other sequence details

Statement in this section for which consensus has been reached:

- Breath-hold scans are not recommended for clinical renal ASL.

## 7.1. Pre and post-inversion saturations are recommended for FAIR labelling schemes.

## 7.2. Background-suppression is recommended for renal ASL.

## 7.3. Renal ASL scans should be performed under free breathing.

## 7.4. Respiratory triggering can be advantageous to minimize the effects of kidney motion at the expense of scan time.

## 7.5. Fat suppression is recommended for renal ASL.

# 8. Data preprocessing

Consensus has been reached for all statements in this section:

- Retrospective image registration is highly recommended for renal ASL.
- Outlier rejection is recommended for renal ASL.

# 9. Quantification

Statements in this section for which consensus has been reached:

- M0 acquisition is mandatory.
- Using a single-compartment model with assumed blood T1 for quantification is recommended for robustness and simplicity.
- Assumed blood T1 at 3T = 1.65s.
- Assumed blood T1 at 1.5T = 1.48s.
- Labelling efficiency PASL = 95% (neglecting background suppression loss).
- Labelling efficiency PCASL = 85% (neglecting background suppression loss).
- When background suppression is used, the labelling efficiency needs to be adjusted based on the number of background suppression pulses.
- Regions of interest selection should be performed manually as the default approach. Semi-automatic methods may be used if local expertise is available (e.g. using T1 maps) but require extensive validation.
- Region of interest selection should be performed based on the ASL M0 image or a separately acquired structural dataset.

## 9.1. A two-compartment model with separate transit time and tissue T1 measurements is a viable alternative to the single-compartment approach but requires more complex acquisition/analysis methods and therefore is currently not recommended as the default renal ASL approach.

## 9.2. Tissue-blood partition coefficient = 0.9 mL/g [Herscovitch1985, 10.1038/jcbfm.1985.9]

# 10. Data analysis/reporting

## 10.1. Minimum set of parameters to be reported (multiple choice)

- Scanner vendor/model
- Coil type
- Field strength
- Shimming routine
- Fat suppression (if used)
- Labelling strategy (FAIR, pCASL, etc...)
- Inflow time(s)/post-labelling delay(s)
- Labelling duration (if applicable)
- Background suppression (if used)
- Readout pulse sequence
- TR
- TE
- Image orientation
- Field of view
- In-plane resolution
- Slice thickness
- Slice gap (if used)
- Slice ordering
- Number of slices
- Number of averages
- Parallel imaging, type and acceleration factor (if used)
- Partial Fourier (if used)
- Bandwidth
- Echo spacing
- Physiological triggering/gating (if used)
- Quantification model

## 10.2. Cortical renal blood flow values (not whole-kidney) should be reported, separately for left and right kidney.

## 10.3. Summary statistics to be reported (subject-wise) (multiple choice)

- Mean
- Median
- Standard deviation
- Interquartile range
- Full range
- ROI size

## 10.4. Summary statistics to be reported (group-wise) (multiple choice)

- Mean
- Median
- Standard deviation
- Interquartile range
- Full range
- ROI size

## 10.5. Medullary renal blood flow values are not considered reliable with current measurement approaches.
